# CLEC5A/TLR2 bispecific antibody suppresses dengue virus-induced pro-inflammatory cytokines production from macrophages

**DOI:** 10.1186/s12929-026-01272-9

**Published:** 2026-06-29

**Authors:** Sheng-Hsuan Wang, Wei-Tzu Kuo, Dayna Cheng, Pei-Shan Sung, Shin-Ru Shih, Shie-Liang Hsieh, Jen-Ren Wang

**Affiliations:** 1 Department of Medical Laboratory Science and Biotechnology, College of Medicine, National Cheng Kung University, Tainan, Taiwan; 2 Institute of Basic Medical Sciences, College of Medicine, National Cheng Kung University, Tainan, Taiwan; 3 Department of Pharmaceutical Sciences, School of Pharmacy, Taipei Medical University, Taipei, Taiwan; 4 Research Center for Emerging Viral Infections, Chang Gung University, Taoyuan, Taiwan; 5 Biomedical Translation Research Center and Institute of Biomedical Sciences, Academia Sinica, Taipei, Taiwan; 6 Immunology Research Center, National Health Research Institutes, Zhunan, Taiwan; 7 Institute of Clinical Medicine, National Yang Ming Chiao Tung University, Taipei, Taiwan; 8 Department of Medical Research, Taipei Veterans General Hospital, Taipei, Taiwan; 9 Center of Infectious Disease and Signaling Research, National Cheng Kung University, Tainan, Taiwan; 10 National Institute of Infectious Diseases and Vaccinology, National Health Research Institutes, Tainan, Taiwan

**Keywords:** Dengue virus, CLEC5A, TLR2, Pro-inflammatory cytokine, Immunotherapy

## Abstract

**Background:**

Dengue virus (DENV) infection drives pathological inflammation through coordinated activation of pattern recognition receptors (PRRs) in myeloid cells, yet no approved immunomodulatory therapy exists to interrupt this process. CLEC5A and Toll-like receptor 2 (TLR2) have each been implicated in DENV-induced cytokine production, but whether their co-engagement represents a convergent and therapeutically targetable axis remains unclear.

**Methods:**

We define the cytokine landscape of human macrophages infected with all four DENV serotypes and reverse genetics DENV2 (rgDV2) strains carrying NS1 mutations derived from the severe 2015 Taiwan outbreak. We further demonstrate the potential therapeutic effect of bispecific antibodies simultaneously targeting CLEC5A and TLR2—engineered in both IgG1 and IgG4 formats.

**Results:**

Human macrophages infected with all four DENV serotypes, as well as reverse genetics DENV2 (rgDV2), exhibit a consistently dominant pro-inflammatory program driven by CLEC5A-TLR2 co-signaling. Treatment with bispecific antibodies, in both IgG1 and IgG4 formats, potently suppress the production of TNF-α, IL-6, IL-8, and MCP-1 production across all viral strains tested. Notably, the IgG4 format preserves robust inhibitory efficacy while minimizing Fc receptor engagement, thereby offering a rational strategy to mitigate the risk of antibody-dependent enhancement (ADE). This dual-targeting approach also remains effective against NS1 mutant strains linked to enhanced virulence and cytokine storm.

**Conclusion:**

Together, these findings identify CLEC5A-TLR2 co-signaling as a convergent inflammatory axis in dengue pathogenesis and establish bispecific dual-receptor blockade as a mechanistically grounded, ADE-aware immunomodulatory strategy to mitigate cytokine-driven pathology.

**Supplementary Information:**

The online version contains supplementary material available at 10.1186/s12929-026-01272-9.

## Introduction

Dengue virus (DENV) is a positive-sense, single-stranded RNA virus belonging to the family *Flaviviridae*, genus *Orthoflavivirus*, and comprises four distinct serotypes: DENV-1, DENV-2, DENV-3, and DENV-4. It is estimated that 390 million DENV infections occur annually, with 96 million individuals developing symptoms and about 500,000 progressing to severe disease, leading to approximately 22,000 deaths each year [1]. In 2015, DENV-2 infection in Taiwan resulted in 43,000 confirmed DENV cases and 228 deaths, making it the largest outbreak of DENV in Taiwan in decades [2–4]. The genome of DENV is approximately 11 kilobases, encoding three structural proteins—the envelope (E), pre-membrane (M), and capsid (C)—and seven non-structural (NS) proteins: NS1, NS2A, NS2B, NS3, NS4A, NS4B, and NS5 proteins [5]. Several studies have highlighted the critical role of NS1 in dengue pathogenesis [6–9]. NS1 is a multifunctional protein involved in viral replication, dissemination, and evasion of host immune responses [9–11]. A previous study by Hee et al. identified four unique amino acid substitutions in the NS1 protein of the 2015 Taiwan DENV-2 strain: P73Q, H224N, K272R, and D278E [12]. Among these, the NS1-K272R substitution was found to promote the secretion of soluble NS1, which in turn enhances the production of pro-inflammatory cytokines [12].

During DENV infection, activation of immune cells drives elevated pro-inflammatory production, contributing to febrile responses and disease progression [13, 14]. Although the pathogenesis of severe dengue remains incompletely understood, it is widely hypothesized that DENV-induced immune cell over-activation leads to a cytokine storm which contributes to severe dengue progression [15]. This excessive cytokine release increases vascular permeability, further contributing to plasma leakage, dengue shock syndrome, and organ dysfunction [16–18]. Macrophages are among the primary target cells of DENV infection, playing a crucial role in viral replication and dissemination and serve as a major source of pro-inflammatory cytokines during infection [19, 20]. Previous reports have demonstrated significantly elevated levels of tumor necrosis factor-alpha (TNF-α), interleukin-8 (IL-8), IL-6, and monocyte chemoattractant protein-1 (MCP-1) in the serum of DENV-infected individuals [20, 21].

C-type lectin domain family 5 member A (CLEC5A), also known as myeloid DAP12-associating lectin-1 (MDL-1), is predominantly expressed on myeloid cells, including monocytes, macrophages, neutrophils, and dendritic cells [22–24]. Upon DENV binding, CLEC5A induces DAP12 phosphorylation and spleen tyrosine kinase (Syk) recruitment [25], activating downstream signaling cascades of nuclear factor kappa-light-chain-enhancer of activated B cells (NF-κB) and interferon regulatory factor 3/7 (IRF3/7) transcription factors that drive pro-inflammatory cytokines and chemokines production [25, 26]. This signaling drives the production of pro-inflammatory cytokines—including TNF-α, IL-6, and IL-8—as well as chemokines such as MCP-1 and IP-10 (CXCL10) [26]. Toll-like receptor 2 (TLR2) is likewise expressed on monocytes and macrophages and has been shown to directly recognize DENV; its activation similarly leads to NF-κB-mediated inflammatory and antiviral interferon (IFN) responses [27]. Importantly, CLEC5A and TLR2 do not act independently—previous studies have demonstrated the formation of CLEC5A/TLR2 heterocomplexes that further amplify pro-inflammatory responses through NLRP3 inflammasome activation [28, 29]. Furthermore, extracellular vesicles released from DENV-activated platelets have been shown to co-stimulate CLEC5A and TLR2, triggering robust cytokine release from macrophages and neutrophil extracellular trap (NET) formation, thereby linking receptor co-signaling to thrombo-inflammatory pathogenesis [28]. The therapeutic relevance of these receptors has been demonstrated in vivo: administration of anti-CLEC5A monoclonal antibody (mAb) significantly increased survival in DENV-infected stat1⁻/⁻ mice [25], while blockade of both CLEC5A and TLR2 by administrating anti-TLR2 mAb in stat1⁻/⁻clec5a⁻/⁻ mice further improved survival rates up to 90% [28], compared to blockade of CLEC5A alone in DENV-infected stat1^–/–^ mice [25]. Collectively, these findings provide a strong mechanistic rationale for the simultaneous blockade of CLEC5A and TLR2 as a therapeutic strategy against DENV-induced inflammation.

In this study, we first characterized the cytokine response profiles of THP-1-derived M1 macrophages infected with DENV serotypes 1–4 and reverse genetics DENV2 (rgDV2) strains harboring NS1 amino acid substitutions identified in the 2015 Taiwan outbreak strain. We then evaluated the therapeutic potential of newly engineered bispecific antibodies—anti-hCLEC5A/TLR2 bsIgG1 and anti-hCLEC5A/TLR2 bsIgG4-designed to simultaneously block of CLEC5A and TLR2 signaling in DENV-infected macrophages. Our findings reveal that a dominant pro-inflammatory response constitutes a conserved feature of macrophage activation across all DENV strains tested, and that dual CLEC5A/TLR2 blockade markedly suppresses the production of TNF-α, IL-8, IL-6, and MCP-1. Collectively, these results establish simultaneous CLEC5A/TLR2 inhibition as an effective strategy for attenuating DENV-induced macrophage hyperinflammation and provide a mechanistic rationale for the development of bispecific antibody-based immunotherapeutics against severe dengue.

## Materials and methods

### THP-1 derived M1 macrophage differentiation

For M1 macrophage differentiation, THP-1 cells (ATCC: TIB-202) were cultured in RPMI medium supplemented with 10% fetal bovine serum (FBS). THP-1 cells (2 × 10^5^/mL) were seeded in 48-well plates and incubated at 37 °C in complete RPMI medium supplemented with 100 nM phorbol 12-myristate 13-acetate (PMA) and incubated for 24 h. After incubation, cells were washed twice with phosphate-buffered saline (PBS) and 2 mL of fresh complete medium was added to each well, followed by a 48-h resting period. On the fourth day, cells were washed twice with PBS, and fresh complete RPMI medium was added for M0 macrophages. For further M1 macrophage polarization, complete RPMI medium containing 20 ng/mL interferon-gamma (IFN-γ) and 100 ng/mL lipopolysaccharide (LPS) was used, and cells were incubated for an additional 24 h to complete M1 macrophage differentiation.

### FACS analysis

To detect markers of THP-1-derived M1 macrophages. Cells were stained with anti-CD11b APC, anti-CD86 PercpCy 5.5, anti-CD163 BV421, anti-CLEC5A PE, anti-TLR2 BV510. The CLEC5A antibody and corresponding isotype control were purchased from R&D System (R&D System) and the rest of the antibodies were purchased from BD Biosciences (BD Biosciences). FACS analyses were performed on CytoFlex cytometer (Beckman coulter) and analyzed by using FlowJo software v10.10.0 (BD Bioscience).

### Construction of infectious cDNA clones

The DENV-2 (16681 strain, GenBank Accession no.: KU725663.1) was used as template for infectious clone construction. The construction of infectious clones was similar as previously described [30]. In brief, the plasmid pDL-DENV2-EGFP-C60-10062016-A2 was used as template to perform site-directed mutagenesis producing rg viruses containing each amino acid substitution identification from the 2015 Taiwan outbreak. The plasmids were transfected into BHK-21 cells (ATCC: CRL-12071) (8 × 10^5^/well) in 6-well plates using PolyJet in vitro DNA Transfection Reagent (SignaGen) according to manufacturer’s instructions. The rg viruses were harvested at 7–9 days post-transfection. Then, the rg viruses were passaged twice in Vero cells (ATCC: CCL-81) and collected for further experiments.

### DENV infection in macrophage

The macrophage (~ 1 × 10^5^/well) was well differentiated in 48-well plates first. The cells were then infected by DENV serotypes 1–4 and rgDV2 at MOI = 0.1 with a 2-h adsorption period. After viral absorption, cells were washed with PBS and refreshed with new complete RPMI medium with 10% FBS for incubation. The culture supernatant and cell pellet were harvested at indicated time points (24, 48, 72 h). The collected culture supernatant and cell pellet were used for cytokine level detection from macrophage. The viral strains of DENV serotype 1–4 used in our study include DENV-1 (Hawaii strain, GenBank Accession no.: KM204119.1); DENV-2 (16681 strain, GenBank Accession no.: KU725663.1); DENV-3 (H87 strain, GenBank Accession no.: M93130.1); DENV-4 (1–0093 strain) is a clinically isolated strain from a DENV4-infected patient as previous literature reported [31].

### Bispecific CLEC5A/TLR2 antibody blocking assay

In this study, recombinant bispecific antibodies targeting human CLEC5A and TLR2 were evaluated in both IgG1 (anti-hCLEC5A/TLR2 bsIgG1) and IgG4 (anti-hCLEC5A/TLR2 bsIgG4) formats. Two IgG1 clones and one IgG4 clone were generated for following analyses. Briefly, the anti-human CLEC5A and anti-human TLR2 binding arms were identified through phage display screening as fully human single-chain variable fragment (scFv) and were subsequently engineered into a bispecific antibody format using human IgG1 and IgG4 backbones to generate fully human bispecific anti-human CLEC5A/TLR2 antibodies. The anti-hCLEC5A/TLR2 bsIgG1 (clone 1) and anti-hCLEC5A/TLR2 bsIgG4 were generated in-house, and anti-hCLEC5A/TLR2 bsIgG1 (clone 2) was custom manufactured by BioLegend. For the blocking assay, macrophages were pre-incubated with the anti-hCLEC5A/TLR2 bsIgG1 (clone 1) (1 μg/mL), anti-hCLEC5A/TLR2 bsIgG1 (clone 2) (1 μg/mL), and anti-hCLEC5A/TLR2 bsIgG4 antibody (1 μg/mL) at 37 °C for 1 h. Corresponding hIgG1 isotype (BioLegend, Cat. No. 403502), and hIgG4 isotype (BioLegend, Cat. No. 403701) were used in parallel before DENV infection. Cells were infected with DENV at MOI = 0.1 for 2 h, followed by PBS wash and incubation in complete RPMI with 10% FBS. Cultural supernatants were collected at 24, 48, and 72 h for cytokine analysis.

### Cytokine measurement by multiplex immunoassay and ELISA

The level of pro-inflammatory cytokines TNF-α, IL-8, IL-6, MCP-1, IL-1β, and IL-12(p70); anti-inflammatory cytokines IL-10, IL-4, and transforming growth factor-beta (TGF-β); anti-viral factors IFN-γ, IFN-β, and IP-10 in culture supernatant from DENV infected macrophage was measured by Multi-Plex Immunoassay (MPI) performed by Inflammation Core Facility (Institute of Biomedical Sciences, Academia Sinica, Taiwan). Antibody-conjugated magnetic beads were incubated with cytokine-containing samples, followed by washing and incubation with biotinylated detection antibodies and subsequently Streptavidin–Phycoerythrin (PE). Fluorescence intensity of the beads was measured using the Bio-Plex® 200 system (Bio-Rad, USA), and cytokine concentrations were calculated based on standard curves. For heat map construction, Z-score normalization was applied to pro-inflammatory cytokines, anti-inflammatory cytokines, and antiviral factors across mock and DENV-infected groups. Z-scores were calculated by subtracting the mean from each value and dividing by the standard deviation. The resulting Z-scores were subsequently used to generate the heat map. The expression levels of TNF-α, IL-8, IL-6, and MCP-1 from DENV-infected macrophage were measured by ELISA according to the manufacturer’s protocols (ThermoFisher). The concentrations were measured by spectrophotometry at 450 nm.

### Statistical analysis

Graphs of the results were presented as the mean ± standard deviation (SD). Statistical analysis was performed by using two-way analysis of variance (ANOVA). Significance levels are indicated as follows: *p < 0.05, **p < 0.01, ***p < 0.001, ****p < 0.0001 for upregulation; ^#^*p* < 0.05, ^##^*p* < 0.01, ^###^*p* < 0.001, ^####^*p* < 0.0001 for downregulation. All results and statistical calculations were performed using GraphPad Prism Version 9.5.

## Results

### Expression of CLEC5A and TLR2 in THP-1 derived M1 macrophage

**Fig. 1:**
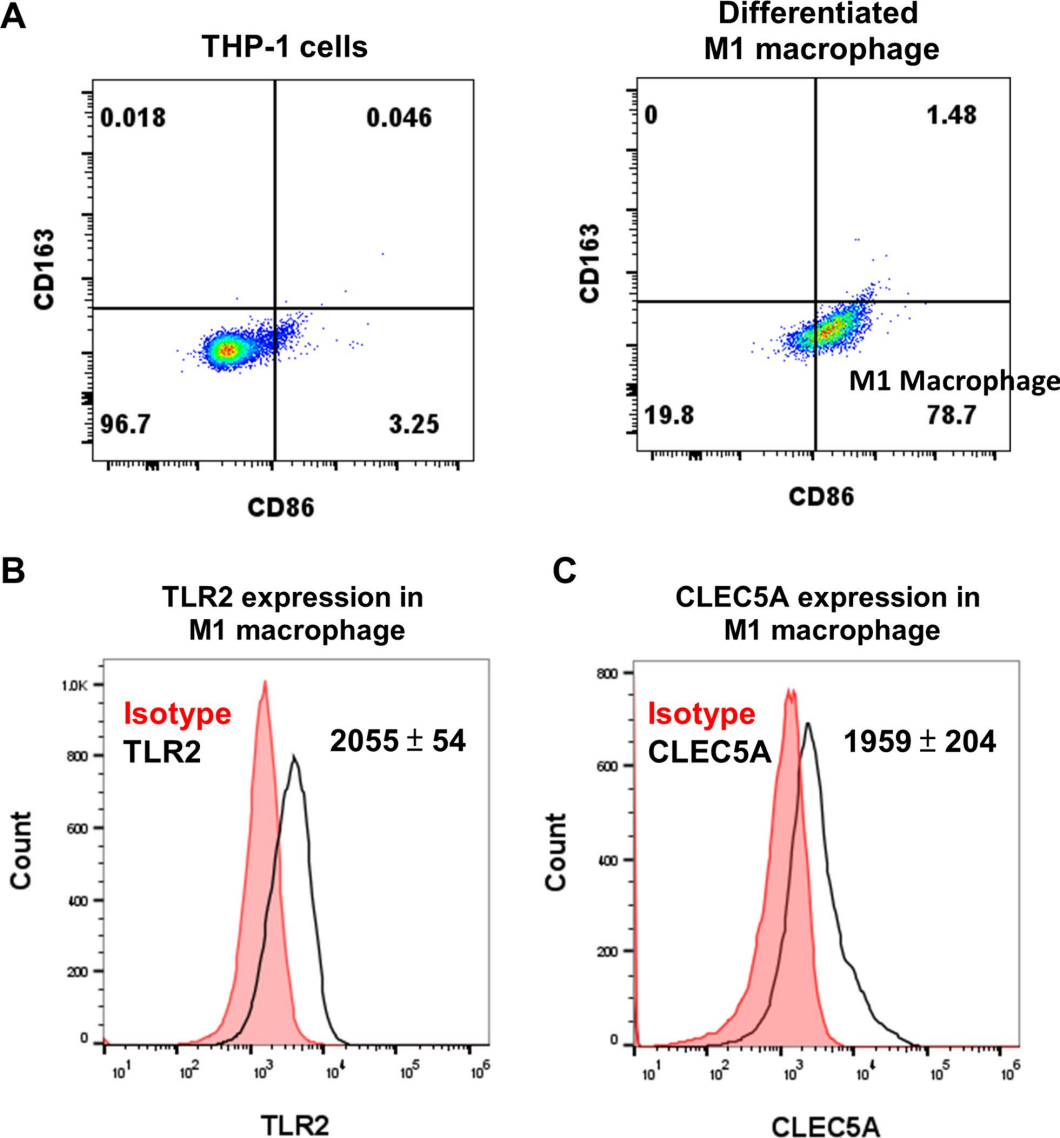
M1 macrophage differentiation and surface TLR2/CLEC5A expression analyzed by flow cytometry. **A** Detection of M1 macrophage polarization from THP-1 cells. The CD86 and CD163 refer to surface marker of M1 and M2 macrophage, respectively. **B**, **C** Expression level of TLR2 (**B**) and CLEC5A (**C**) from THP-1-derived M1 macrophage were determined by FACS analysis. Red area represents isotype control; and black solid lines, indicate mAb of TLR2 and CLEC5A staining. The numbers in the figure show the mean fluorescence intensity (MFI) of mAb staining. The MFI was calculated by FlowJo software and was expressed as the mean ± SD from 3 independent experiments

The THP-1 cell line, a monocyte-derived cell line, is widely utilized in innate immune studies, including studies on M1 macrophages. Initially, we induced differentiation of M1 macrophages from THP-1 cells following established protocols. Flow cytometry was employed to assess the expression of surface markers on THP-1-derived M1 macrophages. CD86 and CD163 were used as markers for M1 and M2 macrophages, respectively. Our findings indicate that a significant proportion of induced cells exhibited high expression of CD86 and low expression of CD163, confirming successful differentiation into M1 macrophages (Fig. 1a). Subsequently, we investigated the expression levels of TLR2 (Fig. 1b) and CLEC5A (Fig. 1c), critical Pattern Recognition Receptors (PRRs) in DENV infection, in M1 macrophages. The mean fluorescence intensity (MFI) of TLR2 (2055 ± 54) and CLEC5A (1959 ± 204) are shown in Fig. 1b, c. Our results demonstrated the expression of both TLR2 and CLEC5A on THP-1-derived M1 macrophages.

### Cytokines profiling by multiplex analysis of DENV-1–4 infected macrophages

**Fig. 2:**
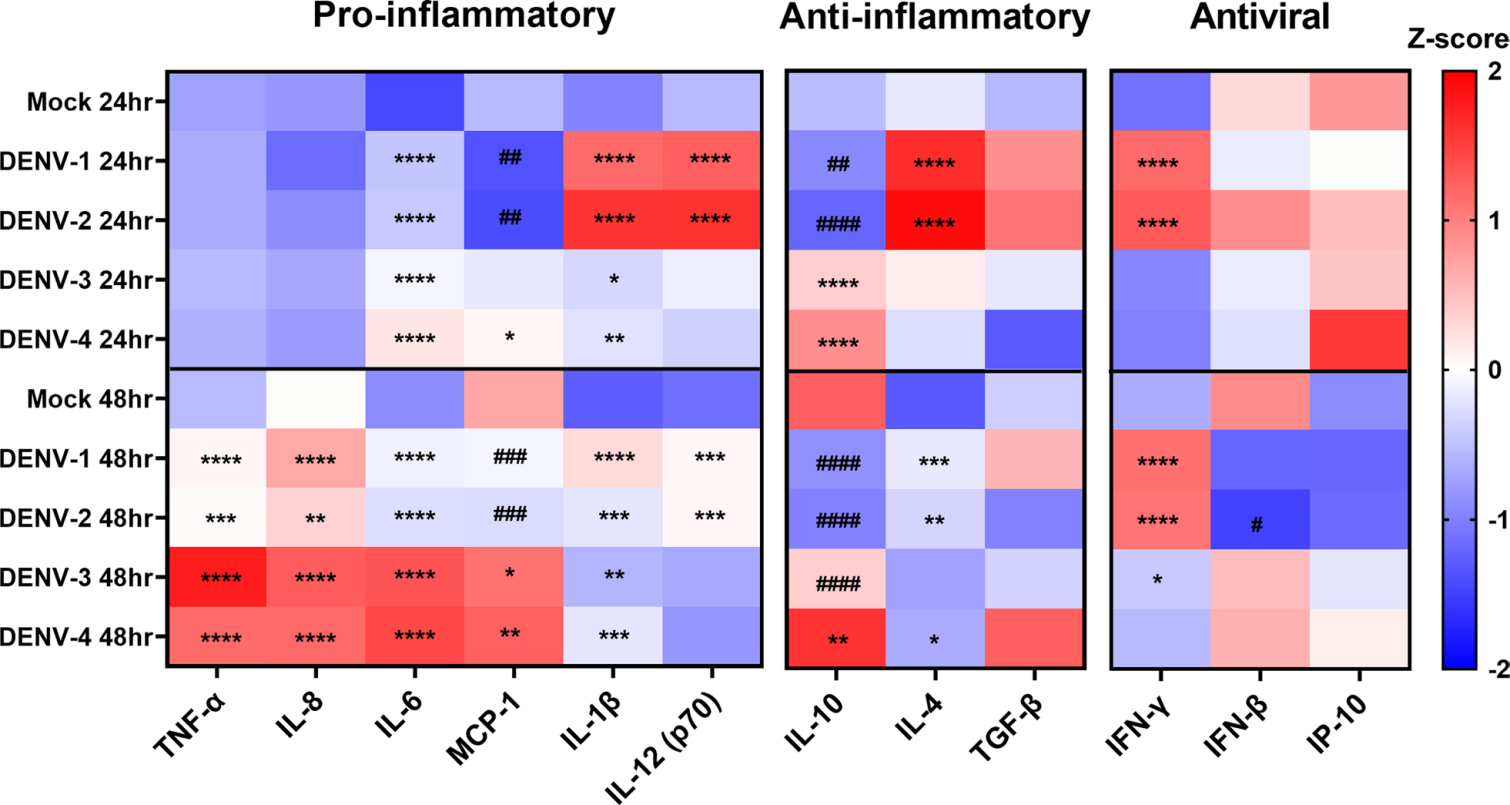
Multiplex analysis of cytokines expression from DENV-1–4 infected macrophages. Heat map showing the expression profiles of pro-inflammatory cytokines, anti-inflammatory cytokines, and anti-viral factors at 24 and 48 h.p.i. Data are presented as Z-score–transformed values. Results represent two independent experiments (n = 2). Significance levels compared to mock are indicated as follows: *p < 0.05, **p < 0.01, ***p < 0.001, ****p < 0.0001 (upregulation); ^#^*p* < 0.05, ^##^*p* < 0.01, ^###^*p* < 0.001, ^####^*p* < 0.0001 (downregulation) by two-way ANOVA

After establishing well-differentiated M1 macrophages, we next investigated the immune responses of macrophages infected with DENV serotypes 1–4 by using multiplex immunoassay. The expression levels of pro-inflammatory cytokines (TNF-α, IL-8, IL-6, MCP-1, IL-1β, IL-12(p70)), anti-inflammatory cytokines (IL-10, IL-4, TGF-β), and antiviral factors (IFN-γ, IFN-β, IP-10) are shown in Fig. 2 and Additional file 1: Fig. S1. Following infection, both TNF-α and IL-8 were significantly upregulated across all four serotypes at 48 h post-infection (h.p.i) when compared with mock infection control. IL-6 was significantly increased in DENV-1–4 infected macrophages at both 24 and 48 h.p.i. MCP-1 expression significantly decreased by DENV-1 and DENV-2 infection but was enhanced by DENV-3 and DENV-4 at 48 h.p.i. The expression of IL-1β was significantly increased across DENV-1–4 infected macrophages, while IL-12(p70) was significantly elevated in DENV-1 and DENV-2 infections at both 24 and 48 h.p.i. Among anti-inflammatory cytokines, IL-10 expression exhibited serotype-dependent patterns, whereby IL-10 was downregulated following DENV-1 and DENV-2 infection, transiently increased at 24 h.p.i but decreased at 48 h.p.i in DENV-3 infection, and markedly upregulated during DENV-4 infection. IL-4 secretion was markedly increased following DENV-1 and DENV-2 infection, and DENV-4 infection also led to elevated IL-4 at 48 h.p.i. Regarding antiviral factors, IFN-γ levels were significantly elevated by DENV-1, DENV-2, and DENV-3 infection, while IFN-β secretion was significantly decreased by DENV-2 infection at 48 h.p.i. Overall, compared with anti-inflammatory cytokines and antiviral factors, pro-inflammatory cytokine secretion from macrophages demonstrated a consistent elevation from 24 to 48 h.p.i. across DENV-1–4, suggesting a dominant pro-inflammatory response during early dengue infection.

### Reduced pro-inflammatory cytokines secretion from DENV-1–4 infected macrophages after CLEC5A/TLR2 bispecific IgG1 antibodies administration

**Fig. 3:**
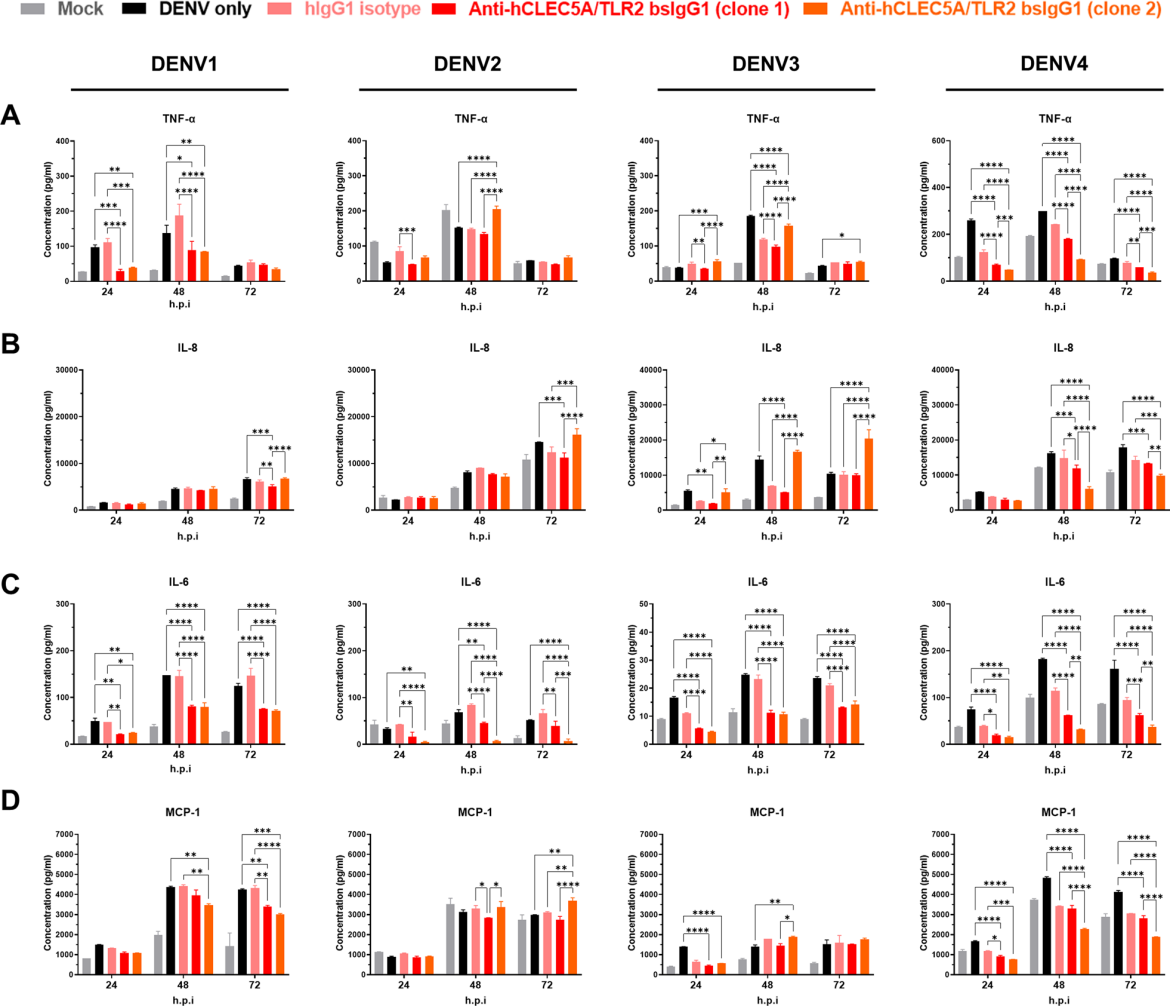
Dual blockade by CLEC5A/TLR2 bispecific IgG1 antibodies reduces pro-inflammatory cytokine expression in macrophages infected with DENV serotypes 1–4. Macrophages were first treated with anti-hCLEC5A/TLR2 bsIgG1 (clone 1), anti-hCLEC5A/TLR2 bsIgG1 (clone 2), and corresponding isotype control (1 μg/mL) for 1 h, then macrophages were infected by DENV-1–4 at MOI = 0.1 for 2 h. Culture supernatant was collected at different times (24, 48, and 72 h) and the concentration of TNF-α (**A**), IL-8 (**B**), IL-6 (**C**), and MCP-1 (**D**) was detected by ELISA. All data are representative data from at least two independent experiments with ****p < 0.0001, ***p < 0.001, **p < 0.01, *p < 0.05 by two-way ANOVA

Both CLEC5A and TLR2 have been identified as critical PRRs involved in the induction of pro-inflammatory cytokine secretion from neutrophils and macrophages. A previous study by Sung et al. further demonstrated that administration of CLEC5A- or TLR2-specific antibodies independently suppressed TNF-α and IL-6 production in DENV2-stimulated macrophages [28]. To further interrogate this inflammatory signaling axis, we employed bispecific anti-CLEC5A/TLR2 monoclonal antibodies in both IgG1 and IgG4 formats (provided by Dr. Shie-Liang Hsieh), designed to simultaneously inhibit CLEC5A- and TLR2-mediated signaling. Macrophages were pre-incubated with the bispecific antibodies prior to infection with DENV serotypes 1–4. The inhibitory efficacy of each antibody was quantified as the inhibition rate (IR) relative to virus-only controls, and statistically significant differences are summarized in Table 1 and Additional file 1: Fig. S2. Our results showed that both bispecific IgG1 antibodies broadly suppressed DENV-induced pro-inflammatory cytokine secretion, although the magnitude of inhibition varied by viral serotype. TNF-α secretion was significantly reduced in macrophages infected with DENV-1, DENV-3, and DENV-4, with IR ranging from 33.0 ± 28.7 to 73.3 ± 0.6% for anti-hCLEC5A/TLR2 bsIgG1 (clone 1), and 14.9 ± 1.4% to 81.6 ± 0.3% for anti-hCLEC5A/TLR2 bsIgG1 (clone 2), while no significant inhibition of TNF-α was observed in DENV-2 infected macrophages (Fig. 3a; Table 1). For IL-8, anti-hCLEC5A/TLR2 bsIgG1 (clone 1) consistently reduced secretion across all four DENV serotypes, with IRs ranging from 23.0 ± 6.9 to 65.9 ± 0.2%. By contrast, clone 2 exhibited a more selective effect, significantly suppressing IL-8 production only in DENV-4 infected macrophages, with IRs ranging from 45.2 ± 4.7 to 61.9 ± 4.3% (Fig. 3b; Table 1). Most notably, both bispecific IgG1 antibodies potently inhibited IL-6 secretion across all four DENV serotypes. Anti-hCLEC5A/TLR2 bsIgG1 (clone 1) achieved IRs ranging from 33.8 ± 8.9 to 73.3 ± 0.9%, whereas clone 2 demonstrated even greater efficacy, with IRs ranging from 39.6 ± 6.3 to 90.2 ± 1.6% (Fig. 3c; Table 1). Similarly, MCP-1 secretion was significantly reduced in macrophages infected with DENV-1, DENV-3, and DENV-4, with IRs ranging from 20.2 ± 0.9 to 67.3 ± 2.0% for clone 1 and 20.8 ± 2.5% to 59.7 ± 0.7% for clone 2. However, no significant reduction in MCP-1 production was observed in DENV-2 infected macrophages following IgG1 bispecific antibody treatment (Fig. 3d; Table 1). Collectively, these findings demonstrate that dual CLEC5A/TLR2 blockade broadly attenuates DENV-induced macrophage inflammatory responses, with the strongest inhibitory effects observed against DENV-4 and comparatively weaker efficacy against DENV-2. These results further support CLEC5A-TLR2 co-signaling as a conserved and therapeutically actionable inflammatory axis in dengue pathogenesis.

**Table 1:** Inhibition (%) of anti-hCLEC5A/TLR2 bsIgG1 antibodies on pro-inflammatory cytokines secretion in DENV1-4 infected macrophages

Antibodies	Anti-hCLEC5A/TLR2 bsIgG1 (clone 1)			Anti-hCLEC5A/TLR2 bsIgG1 (clone 2)		
Cytokines	24 h	48 h	72 h	24 h	48 h	72 h
DENV-1						
TNF-α	71.2 ± 4.4***	33.0 ± 28.7*	N.I.	59.7 ± 1.3**	37.6 ± 10.4**	20.1 ± 3.2
IL-8	22.2 ± 3.1	6.6 ± 3.6	23.9 ± 2.5***	8.6 ± 5.7	0.2 ± 6.4	N.I.
IL-6	56.8 ± 7.1**	45.3 ± 2.0****	39.2 ± 3.5****	50.5 ± 8.3**	45.8 ± 6.0****	42.4 ± 4.2****
MCP-1	27.1 ± 2.1	9.7 ± 5.4	20.2 ± 0.9**	28.0 ± 1.8	20.8 ± 2.5**	29.2 ± 1.8***
DENV-2						
TNF-α	9.6 ± 5.8	12.2 ± 2.2	18.3 ± 1.6	N.I.	N.I.	N.I.
IL-8	N.I.	4.6 ± 2.0	23.0 ± 6.9***	N.I.	10.9 ± 3.4	N.I.
IL-6	51.2 ± 25.7	33.8 ± 8.9**	24.0 ± 18.2	83.8 ± 2.4**	90.2 ± 1.6****	86.7 ± 8.8****
MCP-1	3.2 ± 3.5	9.6 ± 2.7	7.9 ± 5.7	N.I.	N.I.	N.I.
DENV-3						
TNF-α	7.0 ± 0.3	47.1 ± 3.0****	N.I.	N.I.	14.9 ± 1.4****	N.I.
IL-8	65.9 ± 0.2**	65.1 ± 1.5****	4.5 ± 8.2	8.5 ± 14.1	N.I.	N.I.
IL-6	65.5 ± 0.6****	54.6 ± 2.9****	44.4 ± 2.0****	72.6 ± 0.1****	57.0 ± 2.5****	39.6 ± 6.3****
MCP-1	67.3 ± 2.0****	N.I.	N.I.	59.7 ± 0.7****	N.I.	N.I.
DENV-4						
TNF-α	73.3 ± 0.6****	39.4 ± 0.4****	37.9 ± 1.0****	81.6 ± 0.3****	69.1 ± 0.5****	62.1 ± 3.4****
IL-8	40.9 ± 5.3	25.4 ± 9.5***	25.7 ± 3.7***	46.4 ± 0.8	61.9 ± 4.3****	45.2 ± 4.7****
IL-6	73.3 ± 0.9****	65.9 ± 0.8****	61.0 ± 2.5****	79.5 ± 1.5****	82.2 ± 0.4****	77.0 ± 0.1****
MCP-1	44.3 ± 3.7****	31.6 ± 4.0****	31.8 ± 4.3****	54.3 ± 0.5****	53.0 ± 0.2****	54.3 ± 0.6****

Data is shown by mean ± SD (%), the statistical difference is calculated by two-way ANOVA

Anti-hCLEC5A/TLR2 bsIgG1 (clone 1): recombinant bispecific IgG1 targeting human CLEC5A and TLR2; Anti-hCLEC5A/TLR2 bsIgG1 (clone 2): recombinant bispecific IgG1 targeting human CLEC5A and TLR2, custom manufactured by BioLegend

*Significant reduction of cytokines expression level when compared with virus control; N.I: No inhibition, *p < 0.05; **p < 0.01; ***p < 0.001; ****p < 0.0001

### Treatment of novel CLEC5A/TLR2 bispecific IgG4 effectively decreased pro-inflammatory cytokines expression from DENV-1–4 infected macrophage

**Fig. 4:**
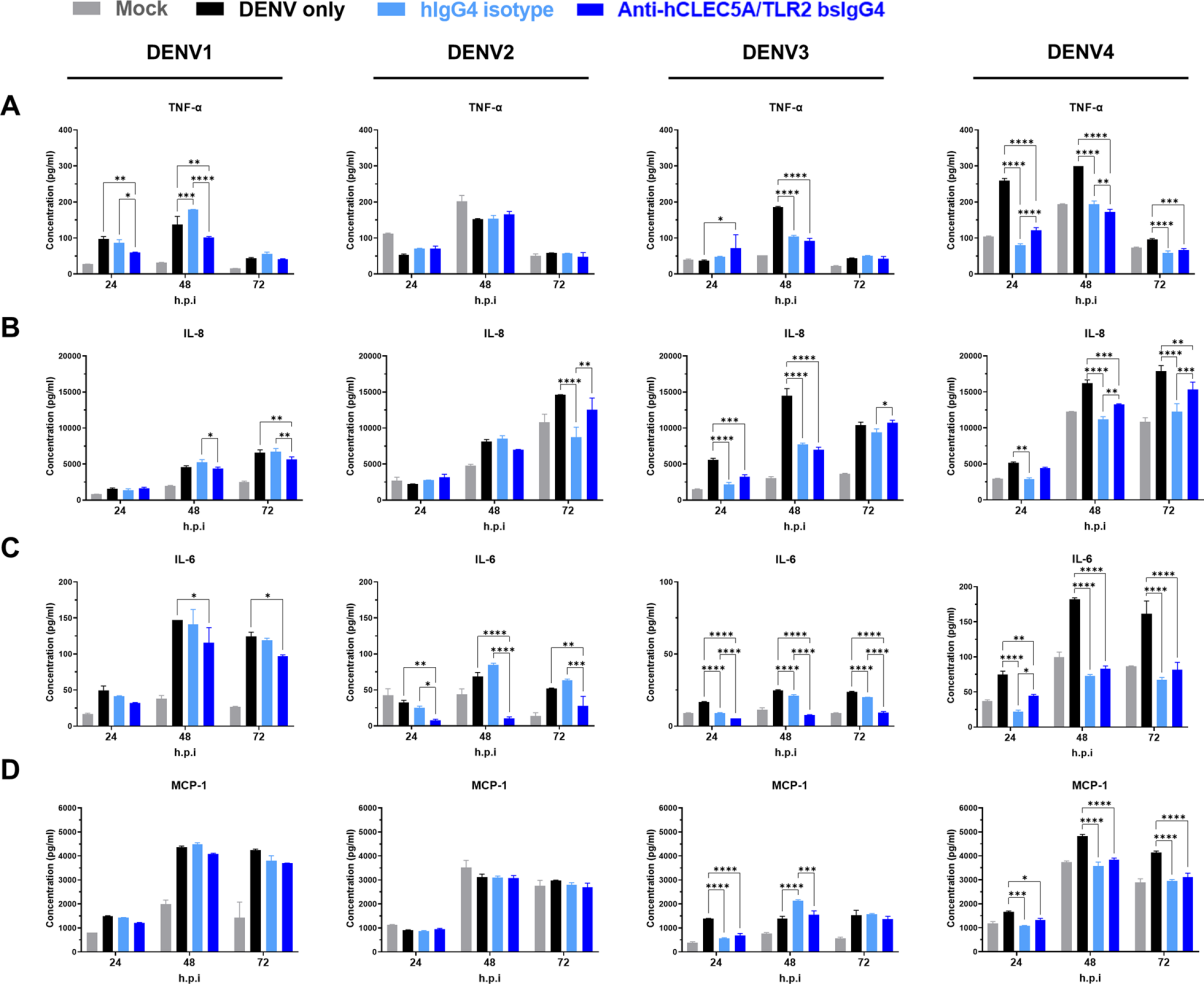
Downregulation of pro-inflammatory cytokine expression in macrophages infected with DENV serotypes 1–4 following CLEC5A/TLR2 blockade by anti-hCLEC5A/TLR2 bsIgG4. Macrophages were first treated with CLEC5A/TLR2 bispecific IgG4 antibodies (1 μg/mL) and corresponding isotype for 1 h, following the infection with DENV1-4 at MOI = 0.1 for 2 h. Culture supernatant was collected at different times (24, 48, and 72 h) and the concentration of TNF-α (**A**), IL-8 (**B**), IL-6 (**C**), and MCP-1 (**D**) was detected by ELISA. All data are representative data from at least two independent experiments with ****p < 0.0001, ***p < 0.001, **p < 0.01, *p < 0.05 by two-way ANOVA

IgG1 antibodies are the principal mediators of systemic humoral immunity, characterized by their potent capacity to activate the classical complement cascade and engage Fc gamma (Fcγ) receptor-mediated effector functions. In contrast, therapeutic antibodies engineered in the IgG4 subclass are specifically designed to minimize the pro-inflammatory effector activities typically associated with IgG1 antibodies, thereby offering a potentially safer immunomodulatory platform. To evaluate whether CLEC5A/TLR2 blockade remains effective in an Fc-effector–attenuated format, we employed anti-hCLEC5A/TLR2 bsIgG4 in macrophage blocking assays following infection with DENV serotypes 1–4, and the IRs are shown in Table 2 and Additional file 1: Fig. S2. Similar to the IgG1 bispecific antibodies, anti-hCLEC5A/TLR2 bsIgG4 significantly suppressed TNF-α secretion in macrophages infected with DENV-1, DENV-3, and DENV-4, with inhibition rates (IRs) ranging from 25.2 ± 10.3 to 53.4 ± 2.0%, whereas no significant inhibition was observed in DENV-2 infected macrophages (Fig. 4a; Table 2). A similar pattern was observed for IL-8 production. Anti-hCLEC5A/TLR2 bsIgG4 markedly reduced IL-8 secretion in macrophages infected with DENV-1, DENV-3, and DENV-4, with IRs ranging from 14.8 ± 0.9 to 61.3 ± 6.8%, while no significant reduction was detected in DENV-2 infected cells (Fig. 4b; Table 2). Notably, IL-6 secretion was robustly suppressed across all four DENV serotypes following anti-hCLEC5A/TLR2 bsIgG4 treatment, with IRs ranging from 21.2 ± 14.1 to 85.0 ± 2.0% (Fig. 4c; Table 2), indicating that IL-6 production remains highly dependent on CLEC5A–TLR2 co-signaling irrespective of viral serotype. For MCP-1, significant inhibition was observed in macrophages infected with DENV-3 and DENV-4, with IRs ranging from 19.6 ± 1.5 to 51.0 ± 6.8%, whereas only modest suppression was detected in DENV-1 and DENV-2 infected macrophages (Fig. 4d; Table 2). Collectively, these findings demonstrate that anti-hCLEC5A/TLR2 bsIgG4 retains substantial anti-inflammatory activity against DENV-induced macrophage activation despite reduced Fc-mediated effector function. The strongest inhibitory effects were consistently observed in DENV-4 infection, whereas DENV-2 exhibited the greatest relative resistance to dual receptor blockade. These results suggest that therapeutic efficacy is primarily driven by direct interruption of CLEC5A–TLR2 inflammatory signaling rather than Fc receptor–dependent mechanisms, supporting the feasibility of an ADE-conscious IgG4-based immunotherapeutic strategy designed to mitigate antibody-dependent enhancement.

**Table 2:** Inhibition (%) of anti-hCLEC5A/TLR2 bsIgG4 on pro-inflammatory cytokines secretion from DENV1-4 infected macrophages

Antibodies		Anti-hCLEC5A/TLR2 bsIgG4							
	Cytokines	24 h	48 h	72 h		Cytokines	24 h	48 h	72 h
DENV-1	TNF-α	40.1 ± 3.7**	25.2 ± 10.3**	5.7 ± 6.3	DENV-3	TNF-α	N.I.	50.2 ± 4.0****	2.0 ± 11.1
	IL-8	N.I.	4.1 ± 0.4	14.8 ± 0.9**		IL-8	61.3 ± 6.8***	46.8 ± 2.1****	N.I.
	IL-6	34.9 ± 6.8	21.2 ± 14.1*	22.4 ± 1.8*		IL-6	67.2 ± 1.0****	68.5 ± 0.3****	59.8 ± 3.5****
	MCP-1	18.3 ± 1.9	6.8 ± 1.9	12.8 ± 0.7		MCP-1	51.0 ± 6.8****	N.I.	8.4 ± 20.2
DENV-2	TNF-α	N.I.	N.I.	17.8 ± 20.5	DENV-4	TNF-α	53.4 ± 2.0****	42.2 ± 2.3****	31.4 ± 5.5***
	IL-8	N.I.	14.1 ± 4.2	N.I.		IL-8	14.7 ± 0.4	16.6 ± 16.2***	14.0 ± 9.2**
	IL-6	77.4 ± 3.7**	85.0 ± 2.0****	46.9 ± 25.2**		IL-6	40.2 ± 1.4**	54.3 ± 2.7****	49.3 ± 0.6****
	MCP-1	N.I.	1.2 ± 7.2	9.9 ± 5.7		MCP-1	19.6 ± 1.5*	20.5 ± 2.4****	24.5 ± 5.2****

Data is shown by Mean ± SD (%), the statistical difference is calculated by two-way ANOVA

anti-hCLEC5A/TLR2 bsIgG4: recombinant bispecific IgG4 antibody targeting human CLEC5A and TLR2

*Significant reduction of cytokines expression level when compared with virus control; N.I: No inhibition, *p < 0.05; **p < 0.01; ***p < 0.001; ****p < 0.0001

### Multiplex analysis of cytokines expression from rgDV2-NS1 mutant viruses infected macrophages

**Fig. 5:**
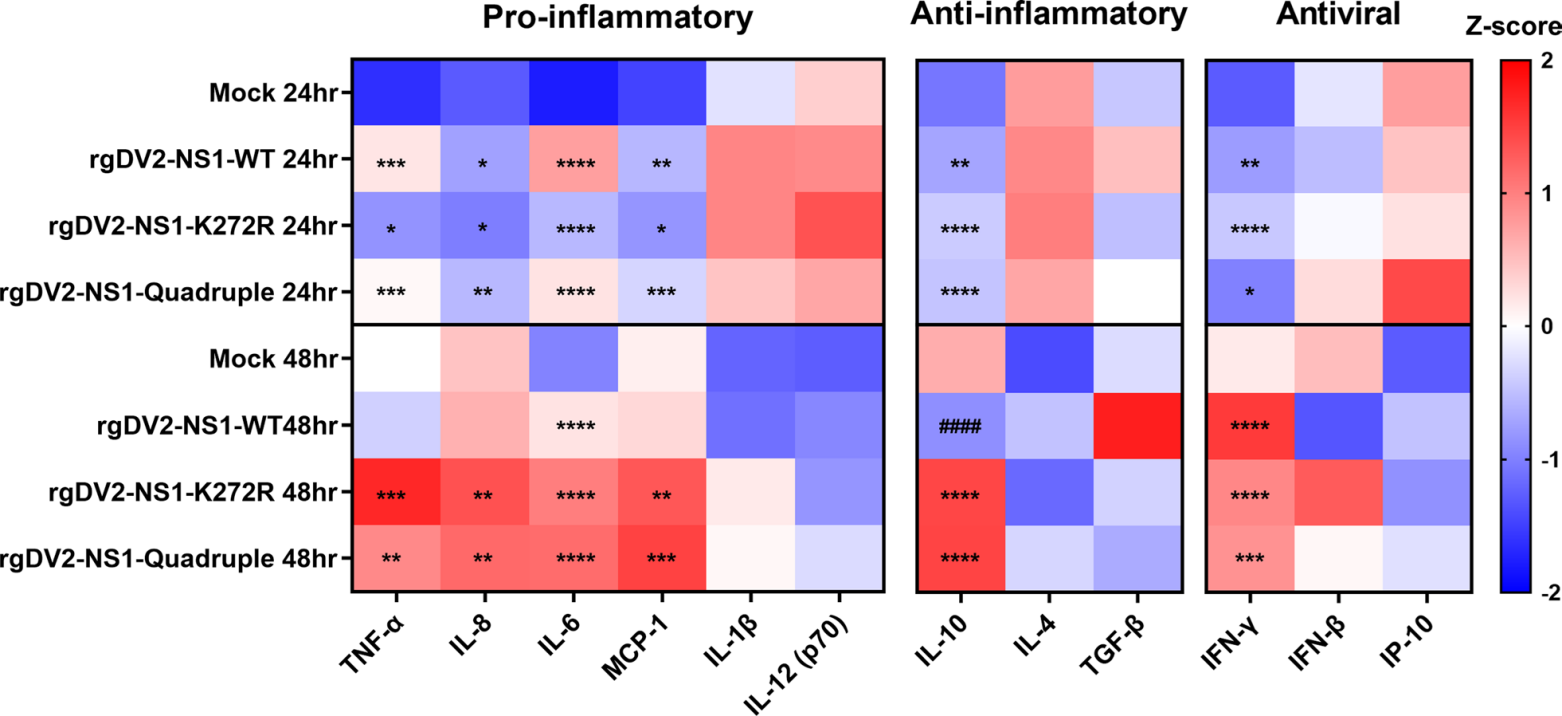
Multiplex analysis of cytokine expression in macrophages infected with rgDV2 viruses. Heat map showing the expression profiles of pro-inflammatory cytokines, anti-inflammatory cytokines, and anti-viral factors at 24 and 48 h.p.i. Data are presented as Z-score transformed values. Results represent two independent experiments (n = 2). Significance levels compared to mock are indicated as follows: *p < 0.05, **p < 0.01, ***p < 0.001, ****p < 0.0001 (upregulation); ^#^*p* < 0.05, ^##^*p* < 0.01, ^###^*p* < 0.001, ^####^*p* < 0.0001 (downregulation) by two-way ANOVA

In 2015, DENV-2 infection in Taiwan caused approximately 43,000 cases and 228 deaths. Hee et al. identified four amino acid substitutions in the NS1 protein of the 2015 Taiwan DENV-2 strain. Among them, the rgDV2-NS1-K272R mutant showed significantly enhanced viral replication and higher IL-6 and IL-8 induction in A549 cells [12]. To further assess these mutations on immune cells, cytokine profiles were examined by multiplex immunoassay in macrophages infected with rgDV2-NS1-K272R and -quadruple mutant strains. Cytokine levels were analyzed and categorized into pro-inflammatory, anti-inflammatory, or anti-viral factors as previously described, and the results were presented in Fig. 5 and Additional file 1: Fig. S3. Following infection, TNF-α, IL-8, IL-6, and MCP-1 level were upregulated in macrophages infected with rgDV2-NS1 viruses. Notably, rgDV2-NS1-K272R and rgDV2-NS1-quadruple mutants induced a prolonged elevation of these cytokines, whereas rgDV2-NS1-WT maintained significantly elevated IL-6 levels up to 48 h.p.i. For anti-inflammatory cytokines detection, IL-10 level was markedly elevated in macrophages infected with rgDV2-NS1-K272R and -quadruple mutants, whereas rgDV2-NS1-WT induced a transient increase at 24 h.p.i that declined by 48 h.p.i. For anti-viral factors, IFN-γ secretion was significantly increased following rgDV2-NS1 virus infection. Overall, infection with rgDV2-NS1-K272R and quadruple mutant strains induced a time-dependent increase in TNF-α, IL-8, IL-6, and MCP-1 secretion. Similar to DENV-1–4 infection, immune responses triggered by rgDV2-NS1 viruses were predominantly characterized by pro-inflammatory cytokine production.

### CLEC5A/TLR2 bispecific IgG1 antibodies showed reduction of pro-inflammatory cytokines in rgDV2-NS1-K272R infection

**Fig. 6:**
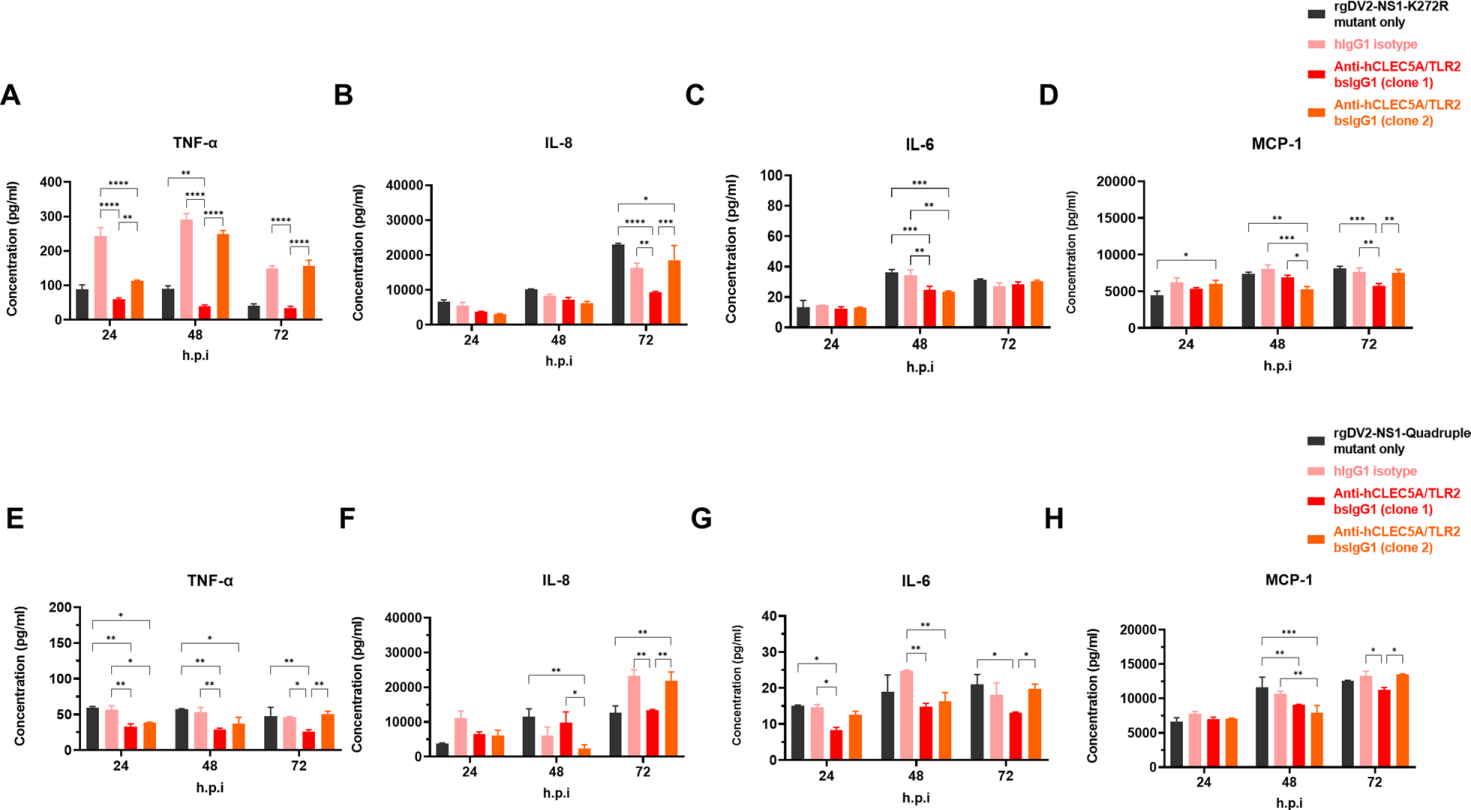
Anti-hCLEC5A/TLR2 bsIgG1 antibodies exhibits superior inhibition of pro-inflammatory cytokine production in macrophages infected with rgDV2-NS1-K272R and rgDV2-NS1-quadruple mutants. Macrophages were treated with anti-hCLEC5A/TLR2 bsIgG1 (clone 1), anti-hCLEC5A/TLR2 bsIgG1 (clone 2) and corresponding isotype control (1 μg/mL) for 1 h and following the infection with rgDV2-NS1-K272R (**A**–**D**) and rgDV2-NS1-quadruple mutant virus (**E**–**H**) at MOI = 0.1 for 2 h. Culture supernatant was collected at different times (24, 48, and 72 h) and the concentration of TNF-α (**A**, **E**), IL-6 (**B**, **F**), IL-8 (**C**, **G**) and MCP-1 (**D**, **H**) was detected by ELISA. All data are representative data from at least two independent experiments with ****p < 0.0001, ***p < 0.001, **p < 0.01, *p < 0.05 by two-way ANOVA

As both CLEC5A/TLR2 bispecific IgG1 antibodies effectively suppressed pro-inflammatory cytokine production in DENV-1–4 infected macrophages, we then evaluated their potential therapeutic effects against rgDV2 harboring NS1 mutations. Macrophages were pre-incubated with the bispecific IgG1 antibodies and subsequently infected with rgDV2-NS1-K272R or quadruple mutant viruses. Culture supernatants were collected at designated time points to measure TNF-α, IL-8, IL-6, and MCP-1 levels, and the IRs are presented in Table 3 and Additional file 1: Fig. S4. During rgDV2-NS1-K272R infection, anti-hCLEC5A/TLR2 bsIgG1 (clone 1) strongly inhibited TNF-α secretion at 48 h.p.i. (IR = 55.8 ± 7.3%), whereas clone 2 showed no inhibitory effect (Fig. 6a; Table 3). IL-8 expression was significantly reduced at 72 h.p.i. by both antibodies, with anti-hCLEC5A/TLR2 bsIgG1 (clone 1) (IR = 59.3 ± 1.4%) showing stronger inhibition than anti-hCLEC5A/TLR2 bsIgG1 (clone 2) (IR = 19.9 ± 19.6%) (Fig. 6b; Table 3). IL-6 secretion was suppressed by both anti-hCLEC5A/TLR2 bsIgG1 (clone 1) and anti-hCLEC5A/TLR2 bsIgG1 (clone 2) at 48 h.p.i. (IR = 32.0 ± 10.0%; IR = 35.4 ± 1.8%, respectively) (Fig. 6c; Table 3). MCP-1 levels were reduced by anti-hCLEC5A/TLR2 bsIgG1 (clone 1) at 72 h.p.i. (IR = 29.6 ± 6.9%) and by anti-hCLEC5A/TLR2 bsIgG1 (clone 2) at 48 h.p.i. (IR = 27.7 ± 7.3%) (Fig. 6d; Table 3). In rgDV2-NS1-quadruple-infected macrophages, both anti-hCLEC5A/TLR2 bsIgG1 (clone 1) and anti-hCLEC5A/TLR2 bsIgG1 (clone 2) significantly inhibited TNF-α secretion (Fig. 6e). Anti-hCLEC5A/TLR2 bsIgG1 (clone 1) reduced TNF-α levels at 24, 48, and 72 h.p.i. (IR = 43.7 ± 4.1%; 49.3 ± 2.2%; 43.5 ± 20.9%), while clone 2 inhibited TNF-α at 24 and 48 h.p.i. (IR = 34.7 ± 1.3%; 34.9 ± 16.6%) (Table 3). IL-8 secretion was unaffected by anti-hCLEC5A/TLR2 bsIgG1 (clone 1) but was strongly inhibited by clone 2 at 48 h.p.i. (IR = 79.9 ± 5.6%) (Fig. 6f; Table 3). IL-6 secretion was significantly reduced by anti-hCLEC5A/TLR2 bsIgG1 (clone 1) at 24 and 72 h.p.i. (IR = 45.0 ± 5.9%; 37.0 ± 7.4%) but was unaffected by anti-hCLEC5A/TLR2 bsIgG1 (clone 2) (Fig. 6g; Table 3). MCP-1 secretion was significant inhibited by both antibodies at 48 h.p.i., with anti-hCLEC5A/TLR2 bsIgG1 (clone 1) (IR = 20.8 ± 10.5%) and anti-hCLEC5A/TLR2 bsIgG1 (clone 2) (IR = 31.8 ± 0.6%) (Fig. 6h; Table 3). Collectively, these findings demonstrate that anti-hCLEC5A/TLR2 bsIgG1 (clone 1) exhibits broader inhibitory efficacy than anti-hCLEC5A/TLR2 bsIgG1 (clone 2) in suppressing pro-inflammatory cytokine production in macrophages infected with rgDV2-NS1-K272R and quadruple mutant strains.

**Table 3:** Inhibition (%) of anti-hCLEC5A/TLR2 bsIgG1 antibodies on pro-inflammatory cytokine secretion in macrophages infected with rgDV2-NS1 mutants

Antibodies		Anti-hCLEC5A/TLR2 bsIgG1 (clone 1)			Anti-hCLEC5A/TLR2 bsIgG1 (clone 2)		
	Cytokines	24 h	48 h	72 h	24 h	48 h	72 h
rgDV2-NS1-K272R mutant	TNF-α	32.2 ± 5.8	55.8 ± 7.3**	18.1 ± 2.8	N.I.	N.I.	N.I.
	IL-8	43.3 ± 7.1	28.3 ± 6.9	59.3 ± 1.4****	54.6 ± 7.7	39.2 ± 6.6	19.9 ± 19.6*
	IL-6	4.3 ± 2.9	32.0 ± 10.0***	9.3 ± 6.7	N.I.	35.4 ± 1.8***	3.1 ± 4.6
	MCP-1	N.I.	6.3 ± 0.7	29.6 ± 6.9***	N.I.	27.7 ± 7.3**	7.6 ± 9.5
rgDV2-NS1-quadruple mutant	TNF-α	43.7 ± 4.1**	49.3 ± 2.2**	43.5 ± 20.9****	34.7 ± 1.3*	34.9 ± 16.6*	N.I.
	IL-8	N.I.	16.3 ± 10.7	N.I.	N.I.	79.9 ± 5.6*	N.I.
	IL-6	45.0 ± 5.9*	19.2 ± 24.8	37.0 ± 7.4*	17.0 ± 5.9	12.9 ± 8.6	5.6 ± 6.1
	MCP-1	N.I.	20.8 ± 10.5**	10.0 ± 3.4	N.I.	31.8 ± 0.6**	N.I.

Data is shown by Mean ± SD (%), the statistical difference is calculated by two-way ANOVA

Anti-hCLEC5A/TLR2 bsIgG1 (clone 1): recombinant bispecific IgG1 targeting human CLEC5A and TLR2; Anti-hCLEC5A/TLR2 bsIgG1 (clone 2): recombinant bispecific IgG1 targeting human CLEC5A and TLR2, custom manufactured by BioLegend

*Significant reduction of cytokines expression level when compared with virus control; N.I: No inhibition, *p < 0.05; **p < 0.01; ***p < 0.001; ****p < 0.0001

### CLEC5A/TLR2 bispecific IgG4 effectively suppressed pro-inflammatory cytokines expression from rgDV2-NS1 mutant viruses infected macrophage

**Fig. 7:**
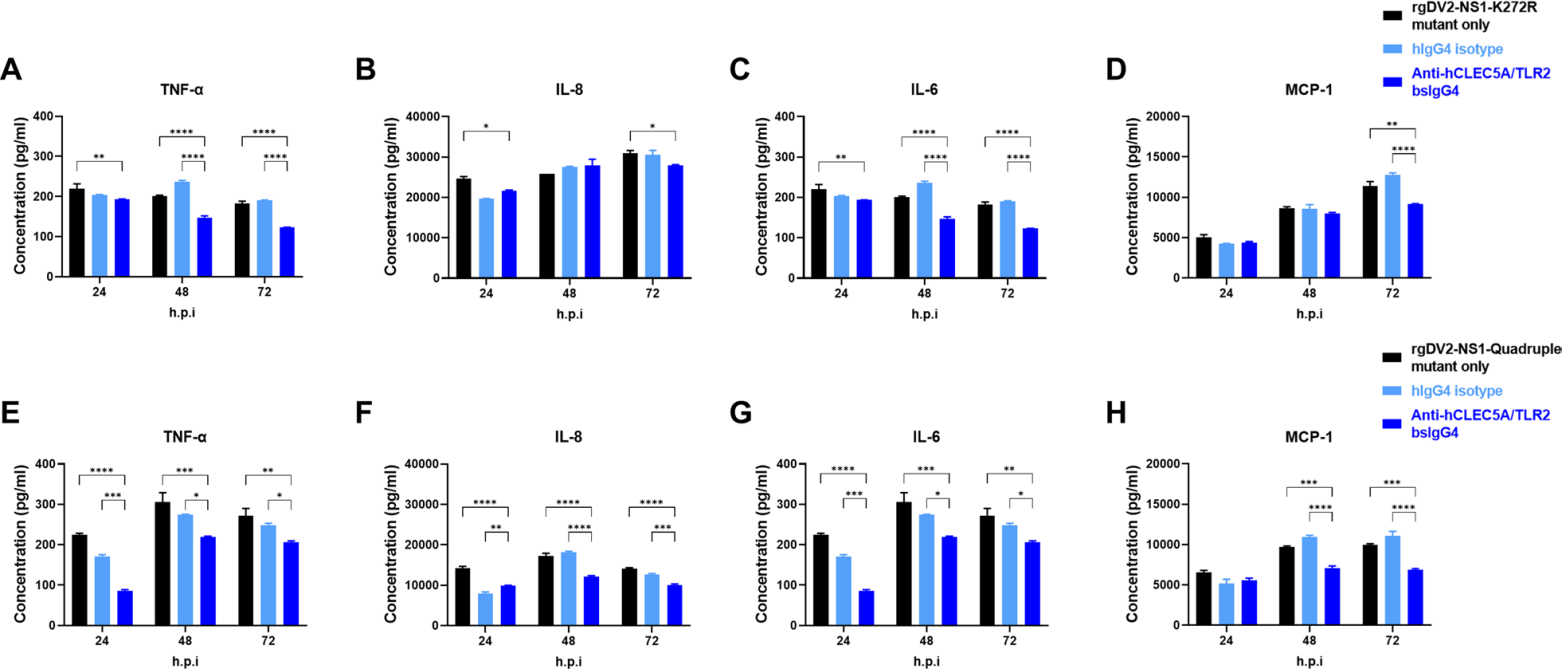
Suppression of pro-inflammatory cytokine expression in rgDV2 mutant-infected macrophages by anti-hCLEC5A/TLR2 bsIgG4. Macrophages were pre-treated with the anti-hCLEC5A/TLR2 bsIgG4 or the corresponding isotype control (1 μg/mL) for 1 h, followed by infection with rgDV2-NS1-K272R (**A**–**D**) and rgDV2-NS1-quadruple mutant virus (**E**–**H**) at MOI of 0.1 for 2 h. Culture supernatants were collected at 24, 48, and 72 h.p.i., and the concentrations of TNF-α (**A**, **E**), IL-6 (**B**, **F**), IL-8 (**C**, **G**), and MCP-1 (**D**, **H**) were measured by ELISA. All data are representative data from at least two independent experiments with ****p < 0.0001, ***p < 0.001, **p < 0.01, *p < 0.05 by two-way ANOVA

Since anti-hCLEC5A/TLR2 bsIgG4 inhibits DENV-1–4 induced pro-inflammatory cytokine secretion from macrophages, we further assessed its effect on rgDV2 carrying NS1 mutations. The anti-hCLEC5A/TLR2 bsIgG4 was pre-incubated with macrophages prior to infection with rgDV2-NS1-K272R and quadruple mutant strains, and the inhibition rates are shown in Table 4 and Additional file 1: Fig. S4. The results showed that anti-hCLEC5A/TLR2 bsIgG4 effectively inhibited TNF-α secretion from macrophages at 24–72 h.p.i. in both rgDV2-NS1-K272R (IR = 15.6 ± 6.5%; 20.9 ± 10.8%; 28.1 ± 5.3%), and rgDV2-NS1-quadruple mutant infections (IR = 61.6 ± 2.5%; 27.5 ± 7.3%; 23.4 ± 8.6%) (Fig. 7a, e; Table 4). Treatment with anti-hCLEC5A/TLR2 bsIgG4 also suppressed IL-8 secretion in rgDV2-NS1-K272R infected macrophages at 24 and 72 h.p.i. (IR = 12.1 ± 3.5%; 9.5 ± 2.0%), and in rgDV2-NS1-quadruple mutant infection from 24 to 72 h.p.i. (IR = 30.4 ± 3.2%; 29.5 ± 1.9%; 28.8 ± 1.0%) (Fig. 7b, f; Table 4). A pronounced inhibitory effect of anti-hCLEC5A/TLR2 bsIgG4 was also observed on IL-6 secretion across all three time points in both rgDV2-NS1-K272R (IR = 11.9 ± 6.7%; 26.9 ± 4.6%; 32.3 ± 3.7%) and rgDV2-NS1-quadruple mutant infections (IR = 61.6 ± 2.4%; 27.6 ± 7.4%; 23.5 ± 8.6%) (Fig. 7c, g; Table 4). For MCP-1, anti-hCLEC5A/TLR2 bsIgG4 treatment reduced secretion levels in rgDV2-NS1-K272R-infected macrophages at 72 h.p.i. (IR = 19.2 ± 5.5%) and in rgDV2-NS1-quadruple mutant infections at 48 and 72 h.p.i. (IR = 27.2 ± 2.9%; 30.8 ± 0.4%) (Fig. 7d, h; Table 4). Similarly, anti-hCLEC5A/TLR2 bsIgG4 treatment showed broader and sustained inhibition of pro-inflammatory cytokines secretion from macrophages infected with rgDV2 harboring amino acid substitution in NS1. In conclusion, both anti-hCLEC5A/TLR2 bsIgG1 (clone 1) and anti-hCLEC5A/TLR2 bsIgG4 effectively suppress pro-inflammatory cytokine responses induced by DENV serotypes 1–4 and rgDV2-NS1 mutants, highlighting their potential as therapeutic candidates to mitigate macrophage activation and DENV-associated inflammation.

**Table 4:** Inhibition rate (%) of anti-hCLEC5A/TLR2 bsIgG4 on pro-inflammatory cytokine secretion in macrophages infected with rgDV2-NS1 mutant viruses

Antibodies		Anti-hCLEC5A/TLR2 bsIgG4							
	Cytokines	24 h	48 h	72 h		Cytokines	24 h	48 h	72 h
rgDV2-NS1-K272R mutant	TNF-α	15.6 ± 6.5**	20.9 ± 10.8****	28.1 ± 5.3****	rgDV2-NS1-quadruple mutant	TNF-α	61.6 ± 2.5****	27.5 ± 7.3***	23.4 ± 8.6**
	IL-8	12.1 ± 3.5*	N.I.	9.5 ± 2.0*		IL-8	30.4 ± 3.2****	29.5 ± 1.9****	28.8 ± 1.0****
	IL-6	11.9 ± 6.7**	26.9 ± 4.6****	32.3 ± 3.7****		IL-6	61.6 ± 2.4****	27.6 ± 7.4***	23.5 ± 8.6**
	MCP-1	12.3 ± 10.3	7.5 ± 0.5	19.2 ± 5.5**		MCP-1	14.9 ± 1.0	27.2 ± 2.9***	30.8 ± 0.4***

Data is shown by Mean ± SD (%), the statistical difference is calculated by two-way ANOVA

anti-hCLEC5A/TLR2 bsIgG4: recombinant bispecific IgG4 antibody targeting human CLEC5A and TLR2

*Significant reduction of cytokines expression level when compared with virus control; N.I: No inhibition, *p < 0.05; **p < 0.01; ***p < 0.001; ****p < 0.0001

## Discussion

CLEC5A and TLR2 are essential PRRs that mediate inflammatory responses during pathogen infection. In this study, the expression of both receptors was first confirmed in THP-1-derived M1 macrophages, providing the cellular basis for subsequent functional studies. We then demonstrated that a dominant program characterizes macrophage responses across all four DENV serotypes and rgDV2 strains harboring NS1 mutations from the 2015 Taiwan outbreak. Crucially, simultaneous blockade of CLEC5A and TLR2 by bispecific antibodies significantly attenuated this response, suggesting that co-engagement of both receptors constitutes a convergent and therapeutically targetable inflammatory axis during DENV infection.

To appreciate the value of this bispecific approach, it is important to consider what mono-specific blockade of either receptor alone cannot achieve. Landmark work by Chen et al. demonstrated that anti-CLEC5A mAb treatment significantly improved survival in DENV-infected stat1⁻/⁻ mice, establishing CLEC5A as a critical driver of DENV-induced lethality [25]. However, this mono-specific blockade is inherently incomplete: TLR2 independently recognizes DENV on monocytes and macrophages and activates NF-κB-mediated inflammatory and anti-viral IFN responses through a parallel signaling axis [27]. Critically, CLEC5A and TLR2 do not function as isolated receptors—they physically associate to form heterocomplexes that further amplify pro-inflammatory signaling through NLRP3 inflammasome activation and are co-engaged by extracellular vesicles released from DENV-activated platelets to drive cytokine storm and NET formation [28, 29]. Importantly, the bispecific format confers an additional pharmacological advantage rooted in binding avidity. Because each arm of the tandem anti-CLEC5A/TLR2 bispecific antibody targets a distinct receptor subunit within the same heterocomplex, activated macrophages that co-present CLEC5A and TLR2 as a physical heterocomplex permit simultaneous bivalent engagement by a single bispecific molecule—one arm anchoring to CLEC5A and the other to TLR2 within the same complex. This cooperative, dual-receptor engagement dramatically increases the apparent binding affinity (avidity) of the bispecific antibody on activated macrophages relative to what either arm could achieve alone. In contrast, resting macrophages that have not yet assembled the CLEC5A/TLR2 heterocomplex permit only monovalent engagement of either receptor independently, yielding substantially weaker antibody binding. This activation-dependent avidity effect is an intrinsic property of the bispecific format that mono-specific antibodies cannot replicate, and it provides a mechanistic basis for preferential engagement and blockade of pathogen-activated, inflammation-driving macrophages over resting cells. The residual inflammatory output from the unblocked receptor is sufficient to sustain significant cytokine production. Indeed, Sung et al. showed that while mono-specific anti-CLEC5A or anti-TLR2 antibodies each independently suppressed TNF-α and IL-6 in DENV-stimulated macrophages, the combined blockade of both receptors—achieved in this study using bispecific antibodies—is required to comprehensively interrupt the synergistic amplification that arises from CLEC5A/TLR2 heterocomplex signaling [28]. The superiority of the bispecific format therefore lies not simply in its dual specificity, but in its ability to disrupt a cooperative signaling node that neither receptor alone can sustain in the absence of its partner. Furthermore, a single bispecific molecule simultaneously occupying both receptor binding sites on the same cell may physically destabilize the heterocomplex itself, an effect that sequential or combined mono-specific antibody treatment may not reliably replicate.

To date, no FDA-approved anti-viral drug exists for the clinical management of dengue, and therapeutic development has predominantly focused on neutralizing antibodies (nAbs) targeting structural viral proteins. Several studies have developed neutralizing antibodies (nAbs) targeting the DENV envelope (E) protein [32–35]. However, the high sequence similarity (72–80%) among the four DENV serotypes means that anti-E nAbs face a fundamental trade-off: antibodies targeting E protein β-barrel domain II (EDII) display cross-reactivity but weak neutralizing potency [36], while those targeting domain III (EDIII) show strong neutralization yet limited serotype coverage [37]. The pre-membrane (prM) protein has also been explored as an antibody target, but prM-reactive antibodies isolated from DENV-infected patients were shown to enhance viral infection in Fc receptor-bearing cells [38]. While recent advances—including Fc-modified anti-prM nAbs that prevent virion maturation and block ADE in vivo [39] and broad-spectrum anti-NS1 mAbs that mitigate endothelial dysfunction [40, 41]—represent meaningful progress, all of these approaches target viral proteins and therefore remain subject to pressure from viral mutation and serotype diversity. The bispecific anti-CLEC5A/TLR2 strategy described here is fundamentally distinct: by targeting conserved host receptors rather than variable viral antigens, it is inherently resistant to viral mutational escape and retains efficacy regardless of serotype—a key advantage confirmed by our data across DENV-1–4 and NS1 mutant strains.

Although the detailed mechanism of severe dengue remained incompletely understood, cytokine storm has emerged as a defining feature of life-threatening disease, making pro-inflammatory cytokine management a compelling therapeutic objective. Prior studies have explored cytokine modulation through pharmacological approaches, including the combination of sunitinib with anti-TNF antibody [42], and natural compounds such as α-mangostin [43] and cordycepin [44], all of which reduce DENV-induced cytokine production by targeting the NF-κB pathway. While these approaches demonstrate proof-of-concept for cytokine-directed therapy, they act downstream of receptor activation and lack the receptor-level specificity to selectively modulate the CLEC5A/TLR2 axis. In contrast, the bispecific antibodies described here act at the upstream receptor level, providing more targeted and mechanistically defined suppression of the CLEC5A/TLR2-driven inflammatory program.

Our results further reveal a serotype-dependent pattern of antibody efficacy. Both bispecific IgG1 formats and the IgG4 format showed the strongest inhibitory effects against DENV-4 and the weakest against DENV-2, consistent with the well-documented higher virulence and greater cytokine-inducing capacity of DENV-2 [45]. Despite the relatively weaker inhibitory effect observed in DENV-2 infection, CLEC5A/TLR2 antibody treatment effectively reduced TNF-α and MCP-1 levels comparable to those induced by DENV-3 and DENV-4 infections, indicating its capacity to suppress excessive inflammatory responses. For most cytokines and antibody formats, DENV-2-induced responses proved more resistant to suppression—with the notable exception of IL-6, which was potently inhibited across all antibody formats and all serotypes, suggesting that IL-6 production in macrophages may be particularly dependent on CLEC5A/TLR2 co-signaling regardless of serotype. Importantly, anti-hCLEC5A/TLR2 bsIgG1 (clone 1) retained the ability to inhibit both IL-8 and IL-6 from DENV-2-infected macrophages where the other formats showed weaker effects, highlighting differential inhibitory profiles that may reflect differences in serotype-specific viral characteristics, host immune activation, and receptor affinity, avidity, or Fc-mediated effector functions between the antibody formats.

The differential efficacy observed against rgDV2-NS1 mutant strains further underscores the therapeutic relevance of this approach. The K272R substitution in the NS1 β-ladder domain enhances soluble NS1 secretion and amplifies pro-inflammatory cytokine induction [12]. Our multiplex data confirmed that both rgDV2-NS1-K272R and the quadruple mutant induced sustained, higher-magnitude cytokine responses in macrophages compared to the wild-type rgDV2, extending the pro-inflammatory findings from epithelial cells [12] to the macrophage compartment most relevant to cytokine storm. Despite this enhanced virulence phenotype, both bispecific IgG1 antibodies and the IgG4 format retained significant inhibitory activity across TNF-α, IL-8, IL-6, and MCP-1, confirming that CLEC5A/TLR2-mediated signaling remains the principal driver of macrophage inflammation even when amplified by NS1 mutations. Notably, anti-hCLEC5A/TLR2 bsIgG4 demonstrated particularly broad and sustained inhibition across all time points and cytokines tested in both mutant strains, suggesting it may be especially well-suited for contexts involving enhanced NS1-driven inflammation.

The choice of antibody subclass carries important translational implications. IgG1 mediates antibody-dependent cellular cytotoxicity (ADCC) and complement activation through Fcγ receptor engagement [46], which may be advantageous for immune activation against infectious agents but raises concerns about ADE in the context of dengue—where Fc receptor engagement by pre-existing antibodies on macrophages facilitates viral entry and amplified infection [38]. IgG4, by contrast, has markedly lower affinity for Fcγ receptors, minimizing ADCC and complement activation [47] and, critically, reducing the risk of Fc-mediated ADE. Although potential Fc-mediated or nonspecific immunomodulatory effects cannot be completely excluded, the inclusion of isotype controls suggest that the observed cytokine inhibition was not caused by nonspecific antibody effects and no obvious abnormal cellular responses or cytotoxic effects were observed under our experimental conditions used. Additionally, our data support that the anti-hCLEC5A/TLR2 bsIgG4 effectively inhibit DENV-induced inflammatory responses, and that the IgG4 backbone represents a more suitable format for further development as an anti-inflammatory therapeutic antibody due to its reduced Fc effector activity. The anti-hCLEC5A/TLR2 bsIgG4 format therefore offers a mechanistically coherent solution to one of the central challenges of DENV antibody therapy: it retains potent receptor-blocking efficacy while structurally mitigating ADE risk through its Fc architecture. This represents a conceptual advance over mono-specific anti-CLEC5A or anti-TLR2 antibodies, which—even if effective as blockers—have not been specifically engineered to address the ADE concern.

The breadth of CLEC5A and TLR2 involvement across diverse pathogens further amplifies the translational significance of these findings. Previous work by Hsieh et al. has established roles for CLEC5A and TLR2 in inflammatory responses to *Listeria monocytogenes*, *Pseudomonas aeruginosa*, Japanese encephalitis virus (JEV), and influenza A virus [24, 48–50], and anti-CLEC5A mAb treatment improved survival in murine models of both DENV and JEV infection [25, 50]. The bispecific format described here may therefore have utility as a broad-spectrum immunomodulatory agent applicable to multiple CLEC5A/TLR2-driven pathologies beyond dengue. Validation in appropriate in vivo models will be essential to fully assess systemic immune modulation, pharmacokinetics, and therapeutic window.

In this study, we utilized THP-1 cells differentiated macrophage as an alternative model for human macrophages since their high reproducibility and stable genetic background. While THP-1-derived macrophages are a well-accepted model for investigating DENV immunopathogenesis, we recognize important limitations of this system. First, cell lines may not fully reflect the complex physiological traits of primary cells. Second, and more specifically relevant to the bispecific antibody strategy described here, THP-1-derived macrophages constitutively co-express CLEC5A and TLR2 following PMA-driven differentiation and may not faithfully recapitulate the dynamic, activation-dependent assembly of the CLEC5A/TLR2 heterocomplex that distinguishes DENV-activated from resting primary macrophages. As a consequence, the activation-dependent avidity advantage of the bispecific antibody—its preferential high-affinity engagement of cells bearing the intact CLEC5A/TLR2 heterocomplex versus comparatively weaker monovalent binding to resting macrophages in which the heterocomplex has not formed—cannot be formally demonstrated in the THP-1 system. Future investigations employing primary human monocyte-derived macrophages, comparing resting and pathogen-activated states, will be essential to substantiate this mechanistic selectivity and further validate the translational potential of our findings.

## Conclusions

**Fig. 8:**
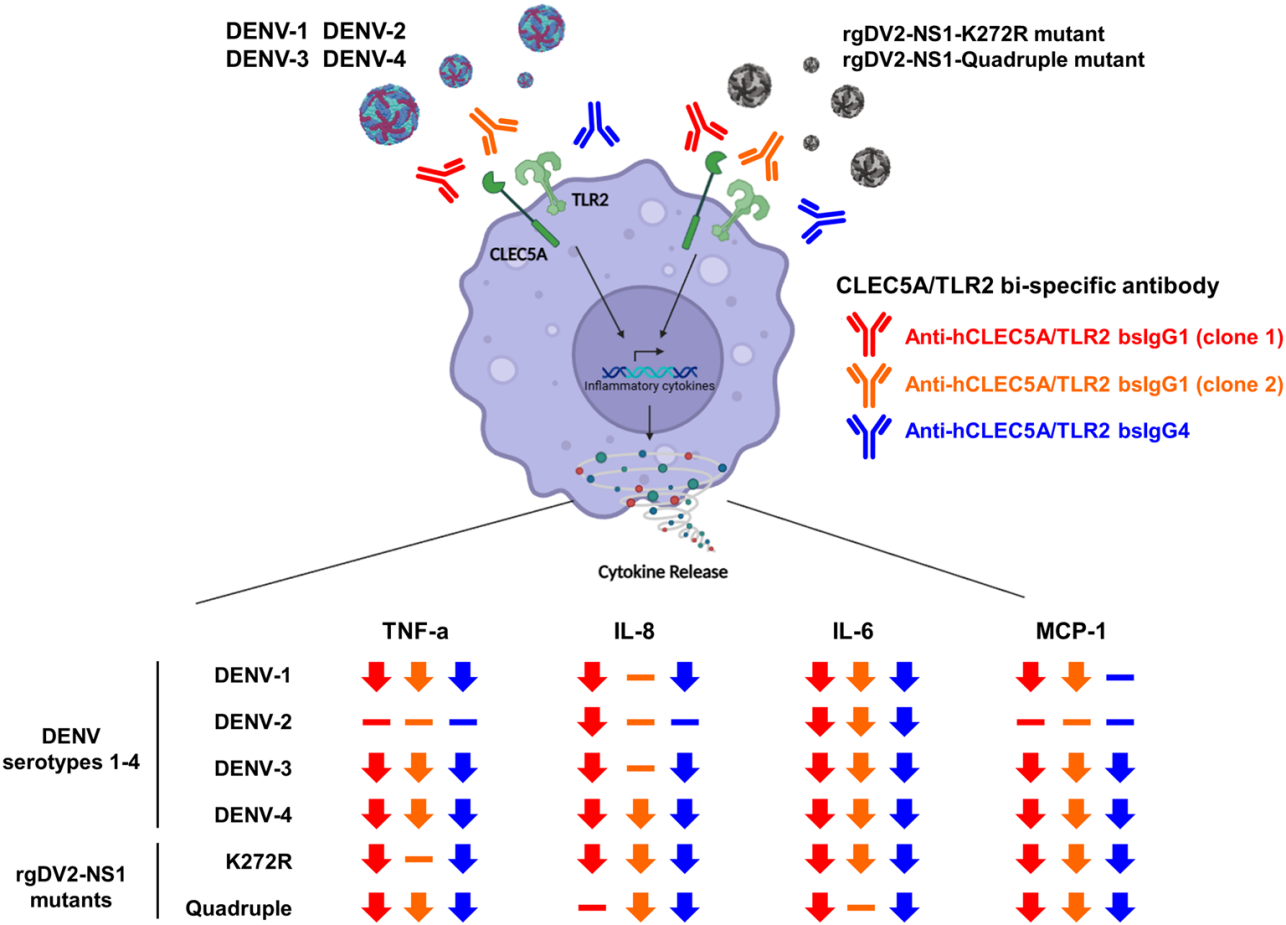
Schematic diagram of bispecific CLEC5A/TLR2 IgG1 and IgG4 antibodies suppress pro-inflammatory cytokines secretion from DENV-infected macrophage. Macrophages infected with DENV serotypes 1–4 and rgDV2-NS1 mutants induce higher pro-inflammatory cytokine production via CLEC5A and TLR2 signaling pathways. Treatment with bispecific anti-hCLEC5A/TLR2 IgG1 or IgG4 significantly reduces pro-inflammatory cytokines secretion from DENV-infected macrophages

To our knowledge, this is the first study to demonstrate the use of bispecific antibodies simultaneously targeting CLEC5A and TLR2, effectively suppressing DENV-induced pro-inflammatory cytokine production in macrophages (Fig. 8). Additionally, we provided a mechanistic rationale as to why this dual blockade was able to achieve what mono-specific receptor targeting could not. By disrupting a cooperative signaling heterocomplex using a host-directed, ADE-aware bispecific antibody strategy, our findings define a new immunotherapeutic framework for attenuating cytokine-driven pathology in severe dengue and establish bispecific CLEC5A/TLR2 antibodies as compelling candidates for further preclinical and clinical development against DENV.

## Supplementary Information

Additional file 1. Fig. S1. Cytokine expression levels in DENV1-4 infected macrophages measured by multiplex immunoassay. Expression level of cytokines, including **A** pro-inflammatory cytokines, **B** anti-inflammatory cytokines, and **C** antiviral factors were detected following DENV1-4 infection at 24 and 48 h.p.i. Significance levels compared to mock are indicated as follows: *p < 0.05, **p < 0.01, ***p < 0.001, ****p < 0.0001 by two-way ANOVA. **Fig. S2**. Inhibition rateof bispecific anti-CLEC5A/TLR2 IgG1 and IgG4 antibodies on pro-inflammatory cytokine secretion in macrophages infected with DENV1-4. Inhibition rate of anti-CLEC5A/TLR2 IgG1 and IgG4 antibodies of DENV1-4 infected macrophages on **A** TNF-α, **B** IL-8, **C** IL-6, and **D** MCP-1. N.I: No inhibition; Significant reduction of cytokines expression level compared with virus control are indicated as follows: *p < 0.05, **p < 0.01, ***p < 0.001, ****p < 0.0001 by two-way ANOVA. **Fig. S3**. Cytokines expression level from rgDV2 mutant virus infected macrophages measured by multiplex immunoassay. Expression level of cytokines, including **A** pro-inflammatory cytokines, **B** anti-inflammatory cytokines, and **C** antiviral factors were detected following rgDV2-WT, rgDV2-NS1-K272R mutant, and rgDV2-NS1-quadruple mutant infection at 24 and 48 h.p.i. Significance levels compared to mock are indicated as follows: *p < 0.05, **p < 0.01, ***p < 0.001, ****p < 0.0001 by two-way ANOVA. **Fig. S4**. Inhibition rateof bispecific anti-CLEC5A/TLR2 IgG1 and IgG4 antibodies on pro-inflammatory cytokine secretion in macrophages infected with rgDV2-NS1 mutant viruses. Inhibition rate of bispecific anti-CLEC5A/TLR2 IgG1 and IgG4 antibodies on TNF-α, IL-8, IL-6, and MCP-1 of **A** rgDV2-NS1-K272R mutant, and **B** rgDV2-NS1-quadruple mutant infected macrophages. N.I: No inhibition; Significant reduction of cytokines expression level compared with virus control are indicated as follows: *p < 0.05, **p < 0.01, ***p < 0.001, ****p < 0.0001 by two-way ANOVA.

## Data Availability

The data supporting the findings of this study can be obtained from the corresponding author upon reasonable request.

## Abbreviations

DENV: Dengue virus
rgDV2: Reverse genetics DENV2
CLEC5A: C-type lectin domain family 5 member A
TLR2: Toll-like receptor 2
TNF-α: Tumor necrosis factor alpha
MOI: Multiplicity of infection
Anti-hCLEC5A/TLR2 bsIgG1: Recombinant anti-human bispecific CLEC5A/TLR2 IgG1
Anti-hCLEC5A/TLR2 bsIgG4: Recombinant anti-human bispecific CLEC5A/TLR2 IgG4
