# Supplementary material for: CLEC5A/TLR2 bispecific antibody suppresses dengue virus-induced pro-inflammatory cytokines production from macrophages
Source: J Biomed Sci. 2026 Jun 29;33:69. doi: 10.1186/s12929-026-01272-9 (PMC13317160; doi:10.1186/s12929-026-01272-9)
Supplement: Supplementary file 1 — Additional file 1. Fig. S1. Cytokine expression levels in DENV1-4 infected macrophages measured by multiplex immunoassay. Expression level of cytokines, including A pro-inflammatory cytokines, B anti-inflammatory cytokines, and C antiviral factors were detected following DENV1-4 infection at 24 and 48 h.p.i. Significance levels compared to mock are indicated as follows: *p < 0.05, **p < 0.01, ***p < 0.001, ****p < 0.0001 by two-way ANOVA. Fig. S2. Inhibition rateof bispecific anti-CLEC5A/TLR2 IgG1 and IgG4 antibodies on pro-inflammatory cytokine secretion in macrophages infected with DENV1-4. Inhibition rate of anti-CLEC5A/TLR2 IgG1 and IgG4 antibodies of DENV1-4 infected macrophages on A TNF-α, B IL-8, C IL-6, and D MCP-1. N.I: No inhibition; Significant reduction of cytokines expression level compared with virus control are indicated as follows: *p < 0.05, **p < 0.01, ***p < 0.001, ****p < 0.0001 by two-way ANOVA. Fig. S3. Cytokines expression level from rgDV2 mutant virus infected macrophages measured by multiplex immunoassay. Expression level of cytokines, including A pro-inflammatory cytokines, B anti-inflammatory cytokines, and C antiviral factors were detected following rgDV2-WT, rgDV2-NS1-K272R mutant, and rgDV2-NS1-quadruple mutant infection at 24 and 48 h.p.i. Significance levels compared to mock are indicated as follows: *p < 0.05, **p < 0.01, ***p < 0.001, ****p < 0.0001 by two-way ANOVA. Fig. S4. Inhibition rateof bispecific anti-CLEC5A/TLR2 IgG1 and IgG4 antibodies on pro-inflammatory cytokine secretion in macrophages infected with rgDV2-NS1 mutant viruses. Inhibition rate of bispecific anti-CLEC5A/TLR2 IgG1 and IgG4 antibodies on TNF-α, IL-8, IL-6, and MCP-1 of A rgDV2-NS1-K272R mutant, and B rgDV2-NS1-quadruple mutant infected macrophages. N.I: No inhibition; Significant reduction of cytokines expression level compared with virus control are indicated as follows: *p < 0.05, **p < 0.01, ***p < 0.001, ****p < 0.0001 by two-way ANOVA. [file 12929_2026_1272_MOESM1_ESM.docx]

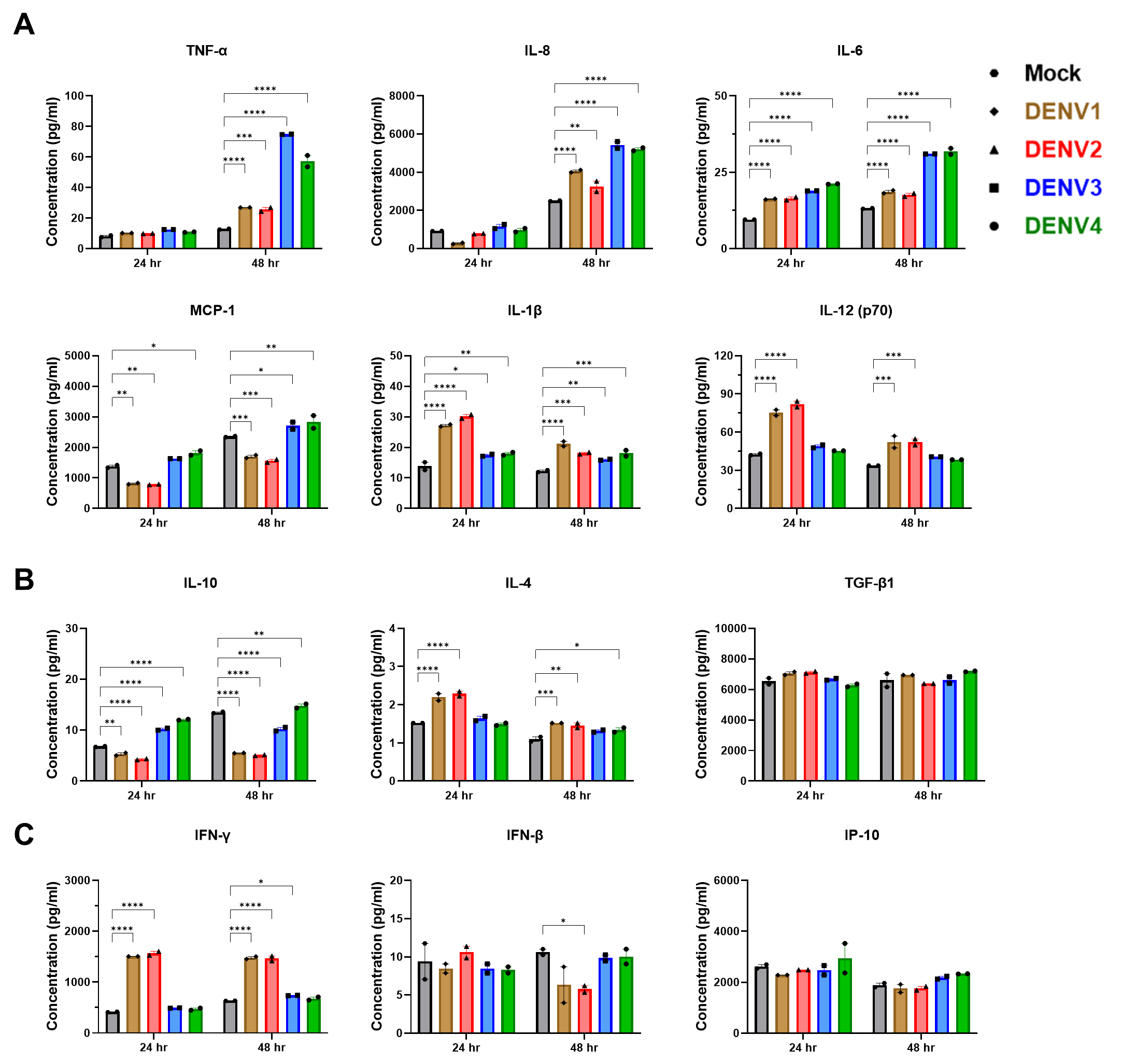


**Fig. S1. Cytokine expression levels in DENV1-4 infected macrophages measured by multiplex immunoassay.**

Expression level of cytokines, including (A) pro-inflammatory cytokines, (B) anti-inflammatory cytokines, and (C) antiviral factors were detected following DENV1-4 infection at 24 and 48 h.p.i. Significance levels compared to mock are indicated as follows: *p < 0.05, **p < 0.01, ***p < 0.001, ****p < 0.0001 by two-way ANOVA.


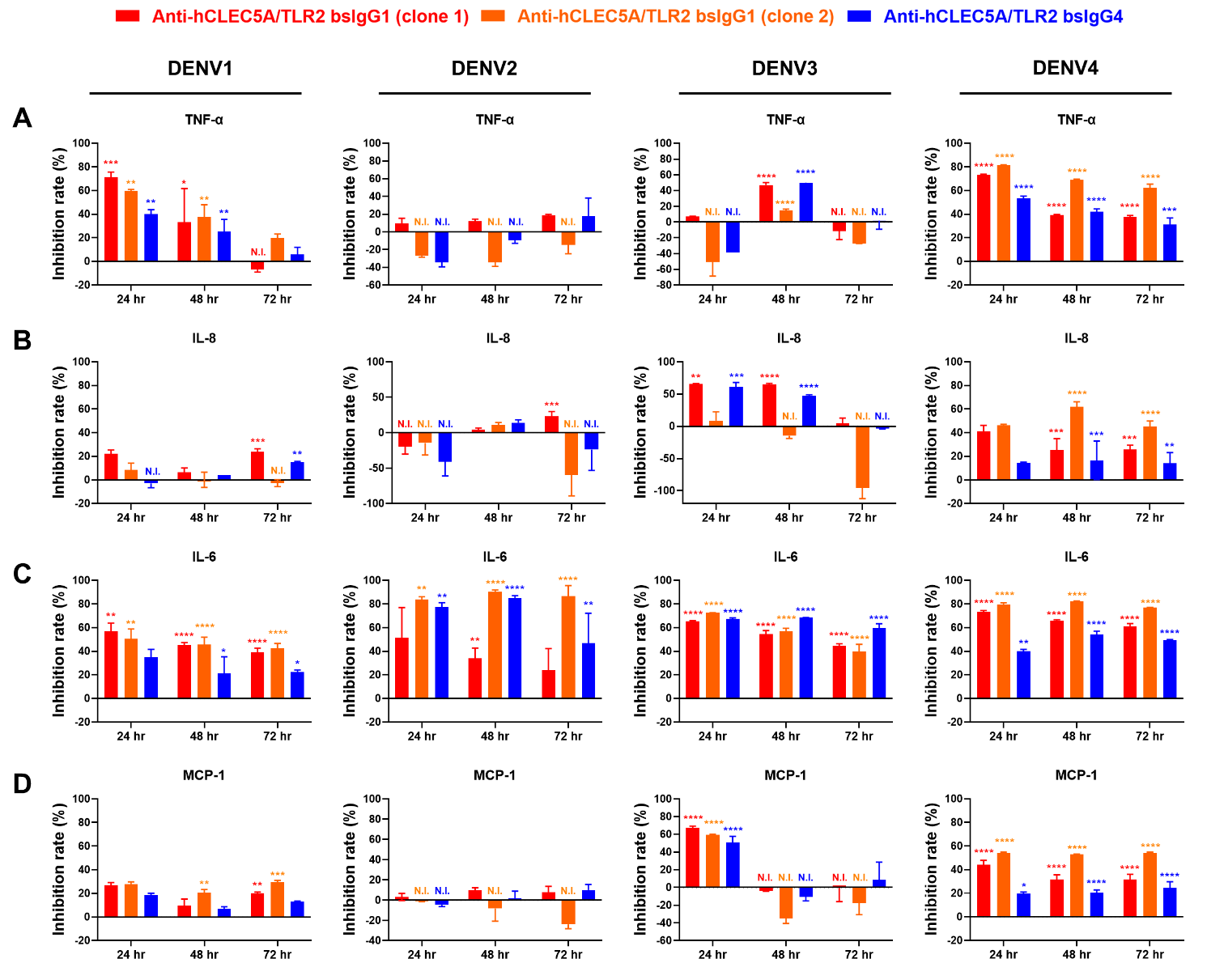


**Fig. S2. Inhibition rate (%) of bispecific anti-CLEC5A/TLR2 IgG1 and IgG4 antibodies on pro-inflammatory cytokine secretion in macrophages infected with DENV1-4.**

Inhibition rate of anti-CLEC5A/TLR2 IgG1 and IgG4 antibodies of DENV1-4 infected macrophages on (A) TNF-α, (B) IL-8, (C) IL-6, and (D) MCP-1. N.I: No inhibition; Significant reduction of cytokines expression level compared with virus control are indicated as follows: *p < 0.05, **p < 0.01, ***p < 0.001, ****p < 0.0001 by two-way ANOVA.


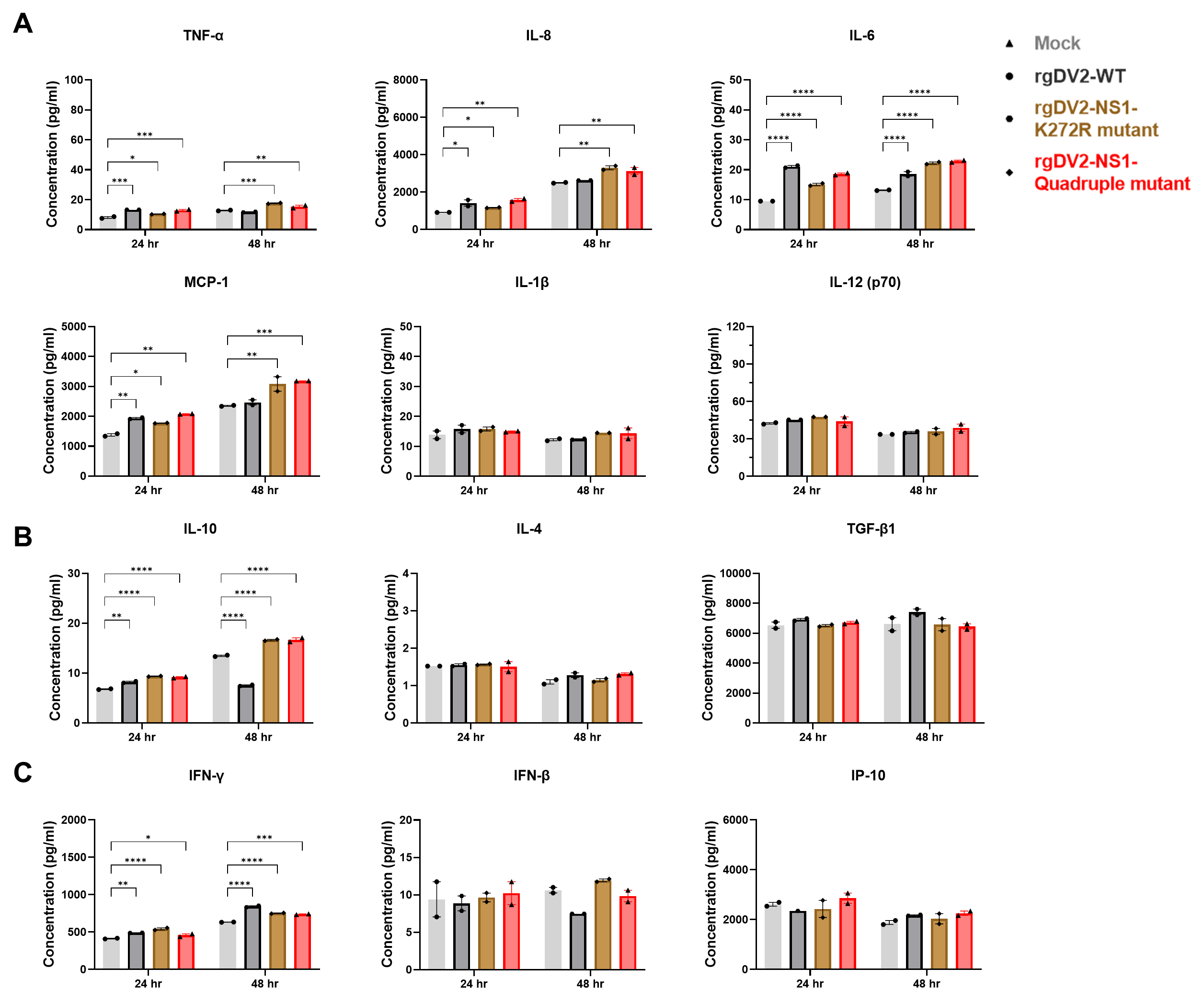


**Fig. S3. Cytokines expression level from rgDV2 mutant virus infected macrophages measured by multiplex immunoassay.**

Expression level of cytokines, including (A) pro-inflammatory cytokines, (B) anti-inflammatory cytokines, and (C) antiviral factors were detected following rgDV2-WT, rgDV2-NS1-K272R mutant, and rgDV2-NS1-Quadruple mutant infection at 24 and 48 h.p.i. Significance levels compared to mock are indicated as follows: *p < 0.05, **p < 0.01, ***p < 0.001, ****p < 0.0001 by two-way ANOVA.

**
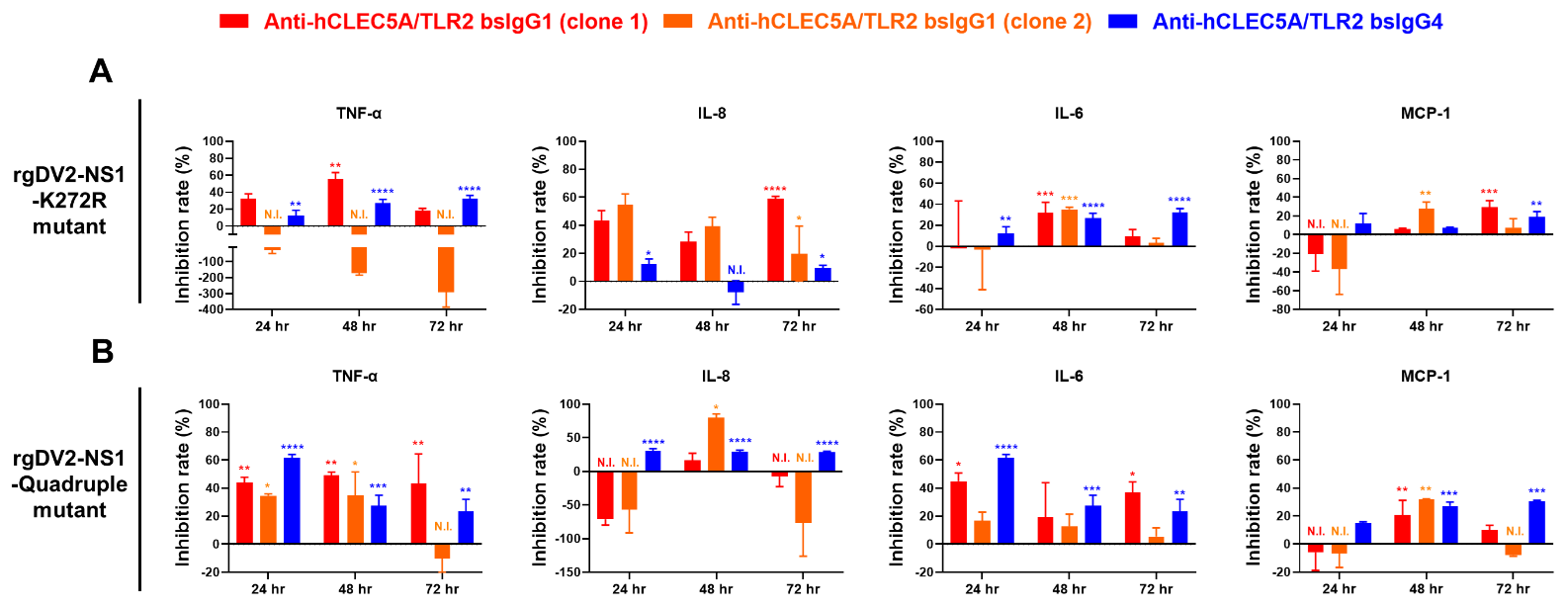
**

**Fig. S4. Inhibition rate (%) of bispecific anti-CLEC5A/TLR2 IgG1 and IgG4 antibodies on pro-inflammatory cytokine secretion in macrophages infected with rgDV2-NS1 mutant viruses.**

Inhibition rate of bispecific anti-CLEC5A/TLR2 IgG1 and IgG4 antibodies on TNF-α, IL-8, IL-6, and MCP-1 of (A) rgDV2-NS1-K272R mutant, and (B) rgDV2-NS1-Quadruple mutant-infected macrophages. N.I: No inhibition; Significant reduction of cytokines expression level compared with virus control are indicated as follows: *p < 0.05, **p < 0.01, ***p < 0.001, ****p < 0.0001 by two-way ANOVA.
